# Rational tuning of high-energy visible light absorption for panchromatic small molecules by a two-dimensional conjugation approach†
†Electronic supplementary information (ESI) available: Experimental details, synthesis and characterization of 2D-**BAI**s, computational details. See DOI: 10.1039/c6sc00428h


**DOI:** 10.1039/c6sc00428h

**Published:** 2016-02-29

**Authors:** Bo He, Danylo Zherebetskyy, Hongxia Wang, Matthew A. Kolaczkowski, Liana M. Klivansky, Tianwei Tan, Linwang Wang, Yi Liu

**Affiliations:** a The Molecular Foundry , Lawrence Berkeley National Laboratory , One Cyclotron Road , Berkeley , California 94720 , USA . Email: yliu@lbl.gov; b Materials Sciences Division , Lawrence Berkeley National Laboratory , One Cyclotron Road , Berkeley , California 94720 , USA; c Beijing Key Lab of Bioprocess , College of Life Science and Technology , Beijing University of Chemical Technology , Beijing 100029 , China

## Abstract

We have demonstrated a rational two-dimensional (2D) conjugation approach towards achieving panchromatic absorption of small molecules. By extending the conjugation on two orthogonal axes of an electron acceptor, namely, bay-annulated indigo (**BAI**), the optical absorptions could be tuned independently in both high- and low-energy regions. The unconventional modulation of the high-energy absorption is rationalized by density functional theory (DFT) calculations. Such a 2D tuning strategy provides novel guidelines for the design of molecular materials with tailored optoelectronic properties.

## Abstract 2

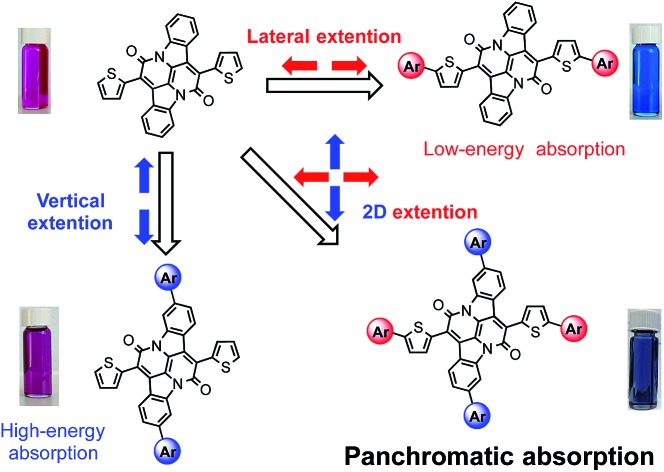
Rational design leads to panchromatic absorption in small molecules by extension of conjugation on two orthogonal axes of an indigo-derived electron acceptor.

## Introduction

Organic semiconductors based on small molecules and conjugated polymers have attracted increasing interest for their applications in clean power generation, such as organic photovoltaics (OPVs)^1^ and dye-sensitized solar cells (DSSCs).^2^ For materials that best harness solar energies, their absorption profiles should have a large overlap with the visible region of the solar irradiance spectrum, which cover more than 60% of the solar photon flux density.^3^ It is therefore essential to design and synthesize single-component panchromatic materials that simultaneously absorb light over multiple visible wavelength ranges. A donor–acceptor approach is among the most rationalized strategies to tune optical properties of organic materials,^4^ which can effectively reduce the optical bandgap towards the lower energy part of the spectrum by linking electron donors and acceptors in a linear conjugated backbone. Such an approach alone, however, is less effective in achieving panchromaticity, as the bathochromic shifts would leave void absorption spaces in the high-energy visible region.^5^ In donor–acceptor conjugated polymers, this situation could be remedied by using two or more different types of co-monomers with sterically hindered side groups. In accordance with the increased bulkiness, the co-planarity of the polymer main chain is decreased, resulting in hypochromic shifts that can fill in the high-energy void.^6^

**Scheme 1:**
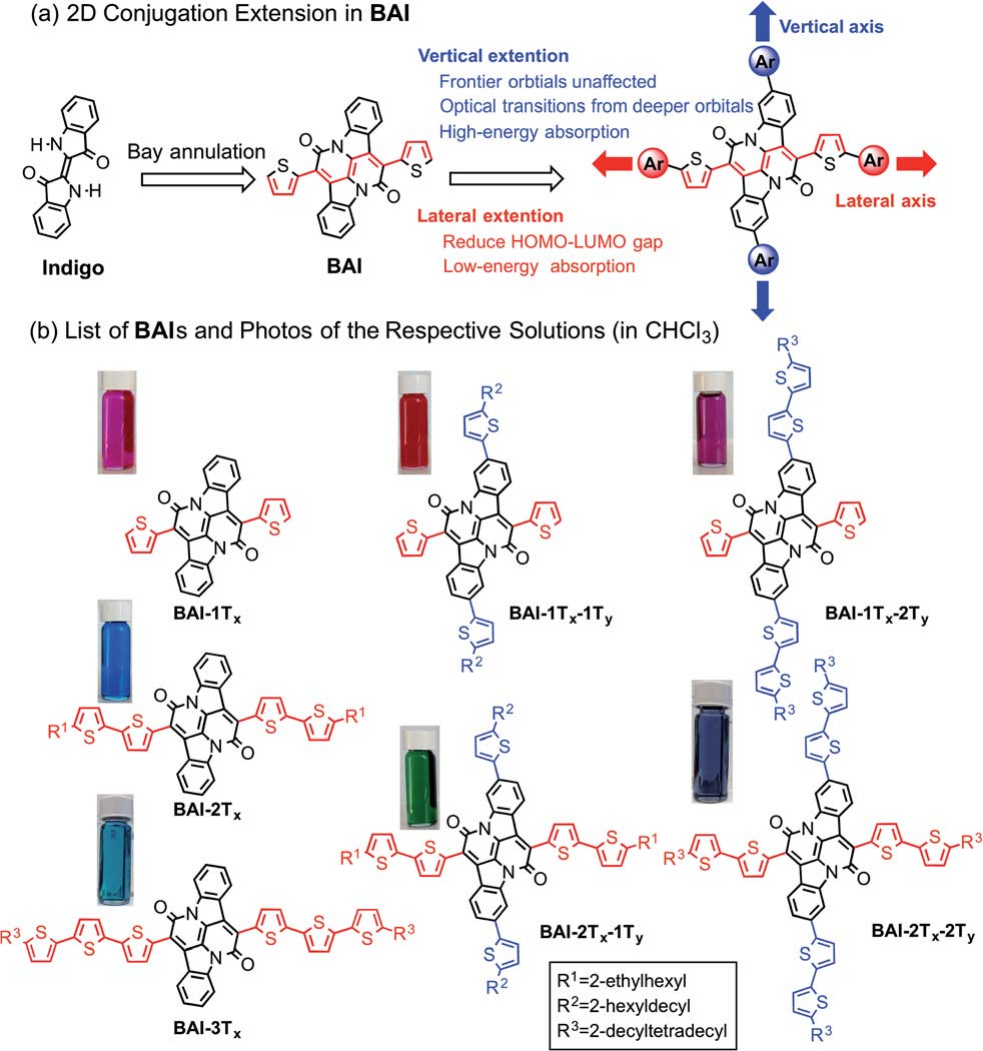
(a) Illustration of 2D extension in **BAI**. (b) Structures of **BAI**s and photos of the respective solutions.

Such a strategy is however not readily applicable for small molecules due to the absence of co-monomer units. As small molecule absorbers are advantageous over polymers in terms of ease of synthesis, less batch-to-batch variations and end-group contamination,^7^ it is imperative to devise a rational design rule that can merge discrete and localized absorptions to give panchromatic small molecules. Despite sporadic reports of small molecules with sub-optimal panchromatic properties,^8^ a rationalized approach is not available.^5^ Here we demonstrate a unique two-dimensional (2D) conjugation approach to afford panchromatic small molecules. The molecular system is based on a recently developed electron acceptor, the bay-annulated indigo (**BAI**) (Scheme 1a).^9^ By simply introducing thiophene units onto two orthogonal directions of a **BAI** core, independent tuning of optical absorptions in both low- and high-energy regions is realized.

Recently, old dyes have resurged as a potent class of building blocks for high performing optoelectronic materials.^10^ New families of π-conjugated systems obtained after chemical transformation of such dye cores have significantly broadened the materials scope of organic semiconductors.^11^ Among these dyes, indigo and related indigoids have received great attention due to their excellent ambipolar charge transport properties.^12^ The direct use of indigo as a building block for organic semiconductors however remains infertile, due to interrupted conjugation across the central double bond and consequently, the π electron resonance is localized within each oxindole unit. The chemical transformation by the double bay annulation step opens up a conjugation pathway that is orthogonal to the original long axis of indigo (Scheme 1a). The resulting **BAI**s behave as excellent electron acceptors, featuring low-lying LUMO energies and narrow optical bandgap.^9^,^13^ As observed in conventional donor–acceptor molecules, further extension of conjugation along the bay-annulation direction effectively lowers the optical bandgap towards the longer wavelength. On the other hand, aromatic units introduced on the orthogonal direction, that is, on the two phenylene rings of the parent indigo, are not fully delocalized and correspondingly induce new absorptions in shorter wavelength region. Theoretical calculations by density functional theory (DFT) have shed light on the nature of 2D electronic coupling and their impact on high-energy absorptions.

## Results and discussion

Scheme 1b lists all the **BAI** derivatives with orthogonally functionalized aromatic substituents. For the ease of annotation, we arbitrarily defined the axis along the bay annulation direction as the “lateral” axis and the orthogonal axis defined by the two phenylene rings as the “vertical” axis. Thiophene-based substituent groups along the lateral or vertical axis of **BAI** were denoted as the suffix to **BAI** as -***n*T*_x_*** or -***n*T*_y_***, respectively, with *n* indicating the number of thiophene units on each side of the **BAI** units. For example, **BAI-2T*_x_*-2T*_y_*** refers to the **BAI** derivative with two thiophene units at each end of the molecules on both lateral and vertical directions. Branched alkyl end groups with longer chain lengths were used in larger **BAI** derivatives to ensure good solubility in common organic solvents.

**Scheme 2:**
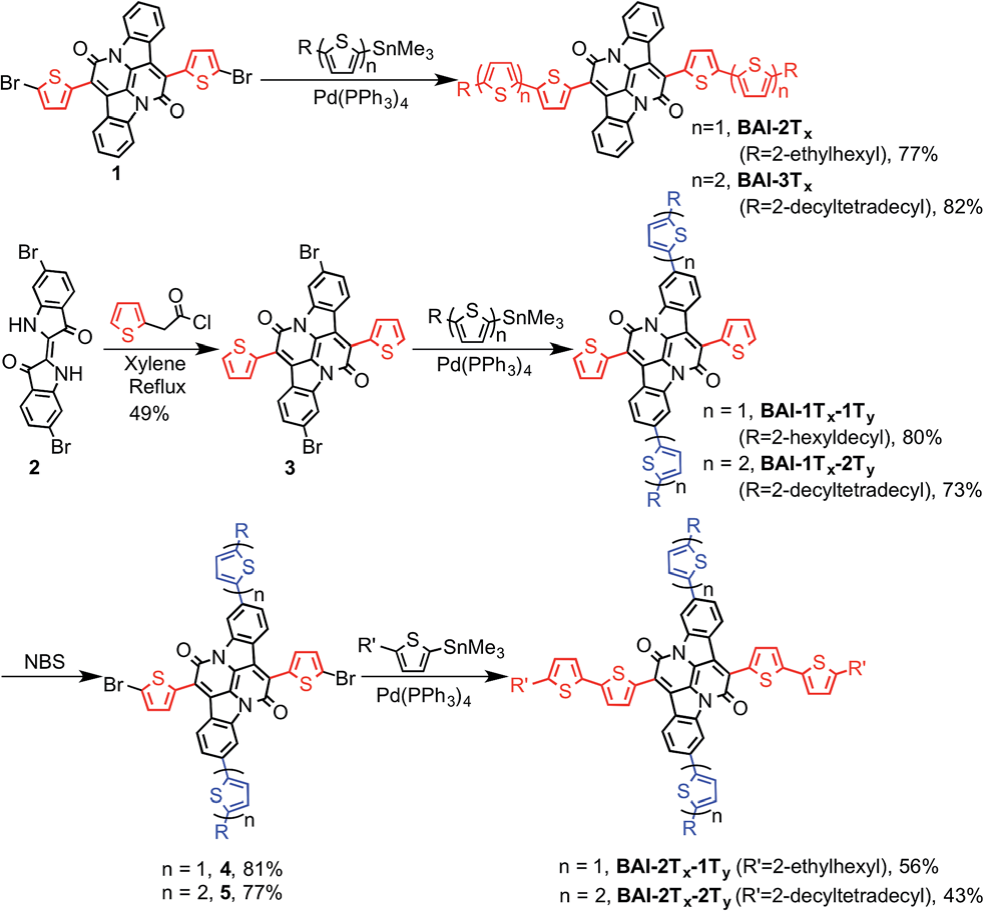
Synthesis of **BAI**-based small molecules.

Lateral extension of **BAI-1T*_x_*** was realized following the previously reported procedures.^9^ As shown in Scheme 2, Stille coupling between dibromide **1**^9^ and oligothiophene stannate gave **BAI-2T*_x_*** and **BAI-3T*_x_*** in good yields. For extension along the vertical axis, **BAI** bromide **3** was first obtained by reacting dibromoindigo **2**^14^ with 2-thienylacetyl chloride in refluxing xylene, which was subjected to Stille coupling to give the corresponding **BAI-1T*_x_*-1T*_y_*** and **BAI-1T*_x_*-2T*_y_***. Bromination of **BAI-1T*_x_*-1T*_y_*** and **BAI-1T*_x_*-2T*_y_***, followed by Stille coupling gave the 2D extended **BAI-2T*_x_*-1T*_y_*** and **BAI-2T*_x_*-2T*_y_***, respectively. The electrochemical properties of all **BAI** compounds were investigated (Fig. S3 in ESI†) by cyclic voltammetry using a conventional three-electrode setup. The frontier orbital energies are estimated and summarized in Table 1 and Fig. S4.† All the **BAI** compounds have a LUMO energy level between –3.53 and –3.69 eV, consistent with their electron accepting properties.

**Table 1:** Summary of optical and electrochemical parameters of **BAI** derivatives

Compd	UV-vis^*a*^					Cyclic voltammetry					
	*λ*_red_ ^*b*^/nm	*ε*/M^–1^ cm^–1^	*λ*_blue_ ^*c*^/nm	*ε*/M^–1^ cm^–1^	*λ*_onset_/nm	*E*_g_ ^*d*^/eV	*E* Ox 1/2 ^,^ ^*e*^/V	*E* Red 1/2 ^,^ ^*e*^/V	*E*_HOMO_/eV	*E*_LUMO_/eV	*E* elec g ^,^ ^*f*^/eV
**BAI-1T*_x_***	579	26 800	—	—	620	1.98	0.58	–1.27	–5.38	–3.53	1.85
**BAI-2T*_x_***	660	47 600	—	—	745	1.66	0.39	–1.13	–5.19	–3.67	1.52
**BAI-3T*_x_***	677	61 700	—	—	800	1.55	0.23	–1.11	–5.03	–3.69	1.34
**BAI-1T*_x_*-1T*_y_***	589	30 900	465	28 800	630	1.97	0.51	–1.24	–5.31	–3.56	1.75
**BAI-1T*_x_*-2T*_y_***	589	21 800	520	25 500	638	1.94	0.47	–1.21	–5.27	–3.59	1.68
**BAI-2T*_x_*-1T*_y_***	669	44 500	494	22 800	749	1.65	0.40	–1.19	–5.20	–3.61	1.59
**BAI-2T*_x_*-2T*_y_***	677	44 300	527	32 000	759	1.63	0.36	–1.12	–5.16	–3.68	1.48

^*a*^In CHCl_3_.

^*b*^Wavelength of lowest energy absorption peak.

^*c*^Wavelength of new high energy absorption peak.

^*d*^Solution optical bandgap.

^*e*^Half-wave potentials (*vs.* Fc/Fc^+^).

^*f*^Solution electrochemical bandgap.

**Fig. 1:**
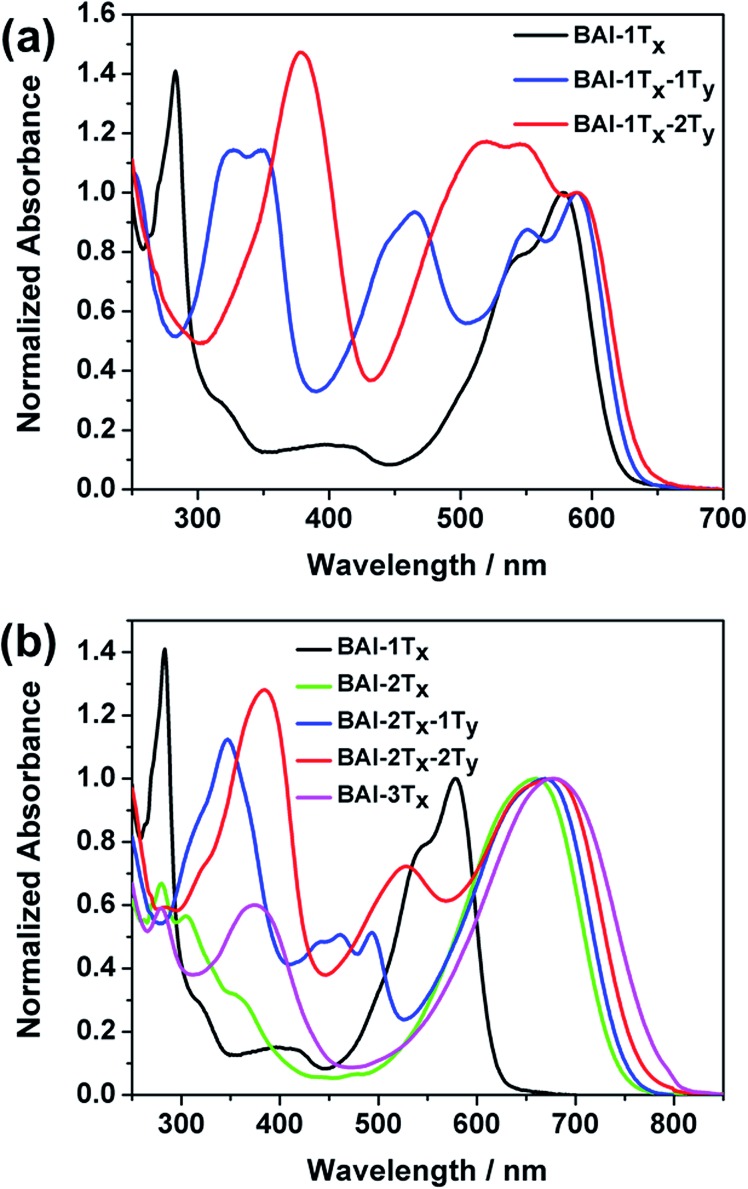
Comparison of UV-Vis absorption spectra of (a) **BAI-1T*_x_*** and the vertically expanded **BAI-1T*_x_*-1T*_y_*** and **BAI-1T*_x_*-2T*_y_***, (b) **BAI-1T*_x_*** and the laterally expanded series **BAI-2T*_x_***, **BAI-2T*_x_*-1T*_y_*** and **BAI-2T*_x_*-2T*_y_***.

The optical absorption spectra of these **BAI** derivatives in CHCl_3_ solutions show distinct correlations in response to extended conjugation in two directions (Fig. 1, absorption normalized against the lowest energy peak, see Fig. S1† for the plots against extinction coefficients). The absorption spectrum of the purple **BAI-1T*_x_*** solution shows double peaks at 542 and 579 nm, characteristic for donor–acceptor molecules that correspond to the π–π* transition and intramolecular charge transfer, respectively.^9^,^15^ The solution of **BAI-1T*_x_*-1T*_y_*** has a reddish-purple color. A comparison of its absorption spectrum against that of **BAI-1T*_x_*** reveals a similar double peak feature, with the lowest energy peak red shifted by 10 nm (Fig. 1a). Notably, a new absorption peak emerges at 465 nm with a similar intensity. In the spectrum of the further vertically extended **BAI-1T*_x_*-2T*_y_*** (Fig. 1a), the lowest energy peak remains at the same position as **BAI-1T*_x_*-1T*_y_***. In the higher energy area, the peak at 465 nm observed in **BAI-1T*_x_*-1T*_y_*** now appears at 520 nm. These spectroscopic changes indicate that vertical extension on **BAI** has a more dramatic effect on the absorption in the higher-energy part of the spectrum.

The impact of lateral conjugation extension on the optical properties is in sharp contrast to the vertical series. For the blue solution of **BAI-2T*_x_*** and **BAI-3T*_x_***, the lowest energy absorptions peak at 660 and 697 nm, respectively, corresponding to red shifts of 81 and 98 nm upon lateral extension of **BAI-1T*_x_*** (Fig. 1b). In comparison to the vertically extended isomer **BAI-1T*_x_*-1T*_y_***, the lateral extension leads to a more remarkable red shift while leaving a virtually void space between 400 and 500 nm in the spectrum. Introduction of one thiophene unit on the vertical direction of **BAI-2T*_x_*** gives **BAI-2T*_x_*-1T*_y_***, the solution of which displays a green colour and a similar lowest energy peak at 669 nm. Encouragingly, new absorption peaks emerge to fill the valley area between 400 and 500 nm. When the vertical conjugation is further extended by installing one more thiophene unit, the resulting **BAI-2T*_x_*-2T*_y_*** exhibits a darkish blue colour in solution. Consistent with the trend observed in Fig. 1a, the vertical extension imposes a small red shift of 8 nm for the lowest energy absorption, while the bluer region absorption becomes more prominent and undergoes a bathochromic shift to 527 nm. Similar features are observed in the absorption spectra of thin films (Fig. S1 and S2 in ESI†), except that peaks are broadened and the absorption onsets are shifted to longer wavelengths with respect to the corresponding solution spectra. The optical characteristics are summarized in Table 1.

Several trends from the optical studies are quite notable: (1) lateral extension of the π conjugation results in significant red shift of lowest energy peaks; (2) vertical substitution with aromatic groups has minimum impact on the bandgap, instead complementary absorption peaks in the bluer region start to emerge; (3) the extinction coefficients of the lowest energy peaks increase as a result of lateral extension of conjugation, but are only slightly affected by vertical expansion. On the other hand, the new absorptions in the high-energy region induced by vertical thiophene substitutions have comparable extinction coefficients (of the order of 10^4^ M^–1^ cm^–1^).

**Fig. 2:**
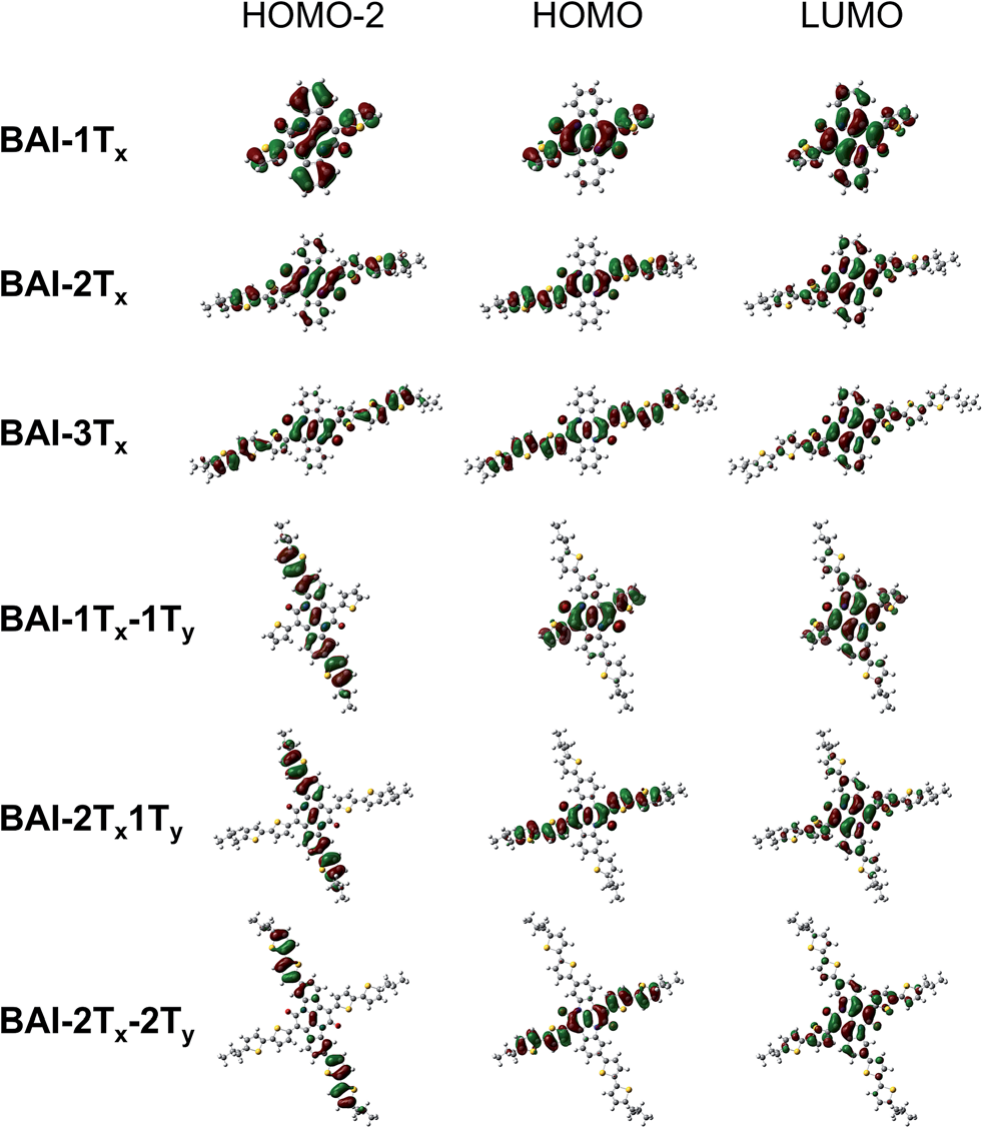
Selected plots of frontier orbitals for the **BAI**s. See Fig. S7† for a more complete set of plots.

In order to understand the correlation between 2D functionalization of **BAI**s and their optoelectronic properties, DFT calculations were carried out to evaluate the frontier orbitals, related energies and the optical transitions. Generally, the lateral extension stabilizes the occupied orbitals and destabilizes the unoccupied orbitals, resulting in decreased energy gaps in between frontier orbitals (Fig. S5†). As shown in Fig. 2, the LUMOs of **BAI-*n*T*_x_*** (*n* = 1–3) are delocalized over the planar aromatic surfaces, including all the thiophenes and the two phenylene rings. The LUMOs are affixed to the lateral conjugation, and are barely affected by any extra thiophene units introduced vertically. On the other hand, confinement of conjugation in the lateral axis is universally observed for all the HOMOs—neither the phenylene rings nor the vertically attached thiophene units have significant contributions to the HOMO orbital. Although vertical functionalization has minimal impact on HOMO and LUMO of these **BAI**s, it effectively stabilizes the deep orbitals (HOMO–1, HOMO–2, *etc*, and LUMO+1, LUMO+2, *etc.*), energetically squeezing them to be close to HOMO and LUMO (Fig. S5†), and rendering optical transitions that are essential for high-energy absorption. The calculated electronic absorption spectra using time-dependent DFT (TD-DFT) show good agreement with the experimental spectra in terms of the relative positions of the absorption bands (Fig. S6†). Calculations on the optical transitions of **BAI**s reveal that while HOMO–1 to LUMO transitions are forbidden with close to zero oscillator strength, HOMO–1 to LUMO+1 transitions are allowed, leading to the intense high-energy peaks (in the 250–420 nm region). For the vertically substituted **BAI**s, optical transitions in the middle range (between the low- and high-energy peaks) are mainly from the HOMO–2 to LUMO transitions, together with smaller contributions from HOMO–4 to LUMO and HOMO to LUMO+2 transitions. More detailed analysis of the compositions of transitions is shown in Table S1.† Fig. 2 compares selected plots of HOMO–2, HOMO and LUMO orbitals of **BAI**s. As indicated, the HOMO–2 orbitals share great similarity to the HOMO orbitals for **BAI-*n*T*_x_*** (*n* = 1–3). When vertical thiophene substituents are introduced, the orbital distributions are drastically altered such that they are confined and delocalized vertically with very little contributions from the lateral aromatic constituents. Due to the alteration of the character of the deep molecular orbitals, it significantly increases (almost order of magnitude) the oscillator strength of the transitions in the center part of the spectrum (Table S1†). Overall, the HOMO–LUMO transition is minimally impacted by vertical extension, while transitions involving the deeper orbitals become the dominant feature in the shorter wavelength region.

## Conclusions

In summary, we have demonstrated a 2D conjugation approach to achieve modular tuning of optical properties of **BAI**-based donor–acceptor molecules. As a result of aromatic substitutions along orthogonal molecular axis, absorptions in both the low- and high-energy regions of the visible spectrum can be finely tuned to achieve panchromatic absorption. Molecular modelling has revealed that the extensions along the lateral and vertical directions impart systematical changes on frontier orbitals and deep orbitals, respectively, which are responsible for the absorptions at different parts of the optical spectrum. The 2D conjugation motif has been applied in the synthesis of cruciform molecules^16^ for sensing^17^ and single-molecule conductance studies,^18^ and the modification of monomers^19^ for fine tuning electronic properties of the resulting polymers, but no insights have been provided towards tuning high-energy absorption profiles. We believe that the underlying principle should provide a more informed search for molecular materials with tailored optoelectronic properties.

## Supplementary Material

Supplementary information
