# Supplementary material for: Rational tuning of high-energy visible light absorption for panchromatic small molecules by a two-dimensional conjugation approach
Source: Chem Sci. 2016 Feb 29;7(6):3857–61. doi: 10.1039/c6sc00428h (PMC6013794; doi:10.1039/c6sc00428h)
Supplement: Supplementary file 1 [file SC-007-C6SC00428H-s001.pdf]

Electronic Supplementary Material (ESI) for Chemical Science.
This journal is © The Royal Society of Chemistry 2016

Electronic Supplementary Information (ESI)

Rational Tuning of the High Energy Visible Light Absorption for Panchromatic Small Molecules by a Two-Dimensional Conjugation Approach

*Bo He, Danylo Zherebetskyy, Hongxia Wang, Matthew A. Kolaczkowski, Liana M. Klivansky, Tianwei Tan, Linwang Wang, Yi Liu**

Table of Contents

Instruments and Methods--Page 1

DFT Calculations-- Page 1

Material Synthesis-- Page 2-6

Table S1-- Page 6

Figure S1--Page 7

Figure S2-- Page 8

Figure S3-- Page 9

Figure S4--Page 10

Figure S5--Page 10

Figure S6--Page 11

Figure S7--Page 12

^{1}H and ^{13}C NMR Spectra--Page 13-21

Instruments and Methods. Dry solvents were collected from a solvent purification system. Thin-layer chromatography (TLC) was carried out using aluminum sheets, precoated with silica gel 60F (Merck 5554). The plates were inspected by UV-light. Proton and carbon nuclear magnetic resonance spectra (^{1}H-NMR and ^{13}C-NMR) spectra were recorded on a Bruker Avance500 II, using locking to the deuterated solvent and using tetramethylsilane as an internal standard. All chemical shifts are quoted using the δ scale, and all coupling constants (J) are expressed in Hertz (Hz). Matrix-assisted laser desorption ionization (MALDI) mass spectra were measured on 4800 MALDI TOF/TOF analyzer from Applied Biosystems. Please note that due to system errors, the high-resolution MS spectra has reflected a high relative error observed in the range between 70 PPM and 150 PPM even after deliberate calibration of the instrument. Cyclic voltammetry was performed using a 273A potentiostat (Princeton Applied Research), wherein glassy carbon, platinum and a silver wire act as the working electrode, the counter electrode and the pseudo-reference electrode, respectively. Samples were prepared in $CHCl_3$ solution with tetrabutylammonium hexafluorophosphate (0.1 M) as the electrolyte at a scan rate of 100 mV s^{-1}, using ferrocene/ferronium (F_c/F_c^{+}) redox couple as an internal standard. The HOMO and LUMO levels of compounds are calculated from the difference between the first oxidation potential (E_{oxi}) or reduction potential (E_{red}) of the compounds and the oxidation potential of ferrocene (E_{HOMO} = -(4.8-E_{oxi}) eV, E_{LUMO}=-(4.8-E_{red}) eV). UV-Vis-NIR spectra were recorded using a Cary 5000 UV-Vis-NIR spectrometer.

DFT Calculations. Ground state geometries of the molecules in the solvent were first optimized using 6-31+G(d)[1] basis set. The optimized geometries were then used for characterization of the low-lying excited states relying on time-dependent DFT (TD-DFT) calculations using 6-311++G(d,p) basis set. Since the experimental measurements were done in chloroform solvent, we used continuum solvation model (SMD) based on the quantum mechanical charge density of a solute[2] that shows more reliable results for prediction of the excitation energy.[3] Additionally, calculations using B3LYP/6-311++G(d,p) give reliable results[4] for molecular orbital eigenvalues after applying the following linear correlation correction.[5]

$$\varepsilon_{LUMO}^{exp} = 1.0729 \cdot \varepsilon_{LUMO}^{calc} - 0.181$$
$$\varepsilon_{HOMO}^{exp} = 1.001 \cdot \varepsilon_{HOMO}^{calc} - 0.050$$

Material Synthesis. 2-thiopheneacetyl chloride was purchased from Aldrich. **1**,[6] **2**,[7] and thienyl trimethyltin compounds[8] were synthesized according to literature. Synthetic procedures towards **3**, **4**, **5** and **BAI-nT$_x$-nT$_y$s** are described as follows.

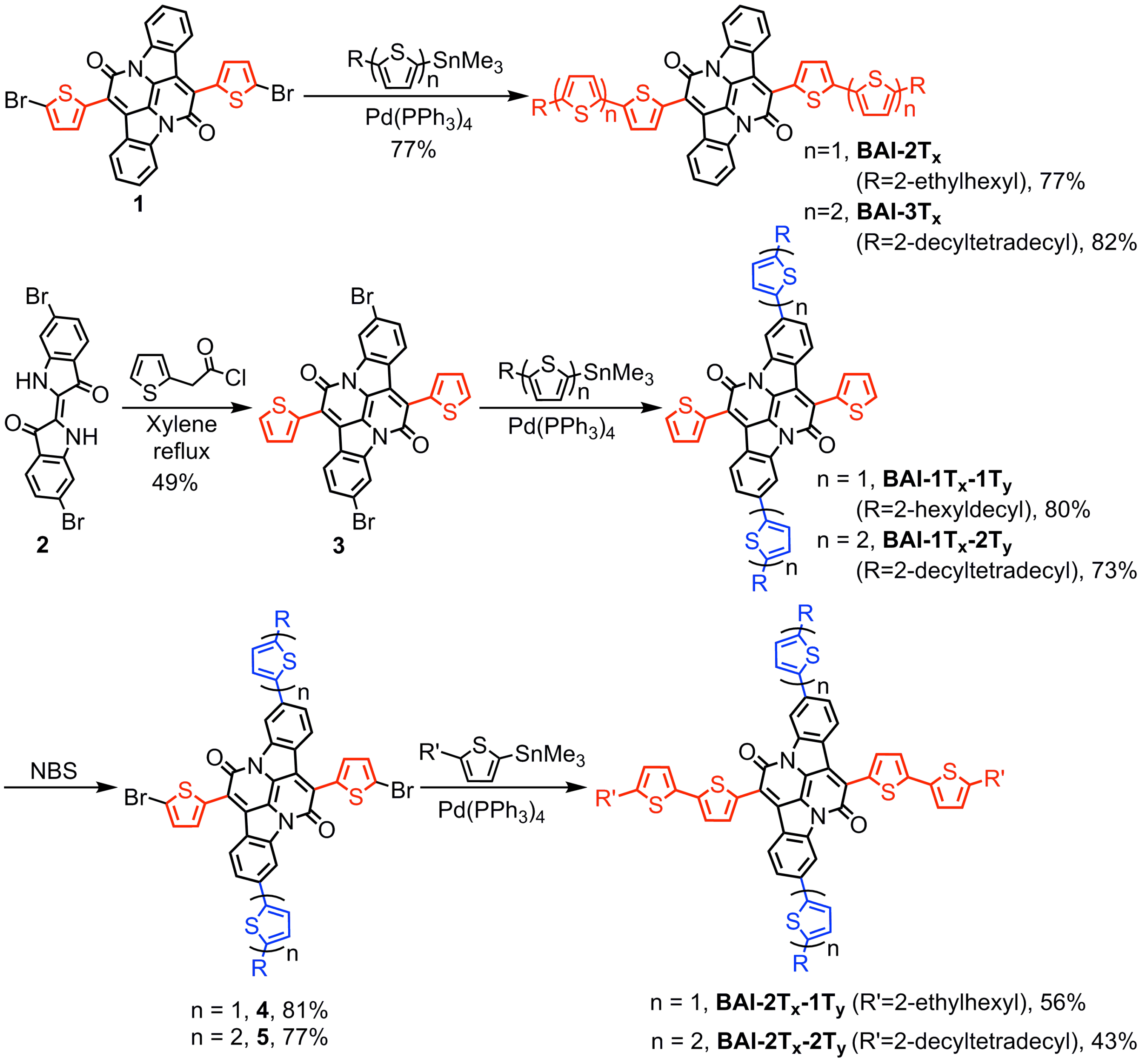

Scheme S1. Synthetic route of the **BAI**s

BAI-2Tx: Under N_2 atmosphere, a deoxygenated mixture of **1** (200 mg, 0.320 mmol, 1.0 eq.), (5-(2-ethylhexyl)thiophen-2-yl)trimethylstannane (254 mg, 0.790 mmol, 2.5 eq.), and $Pd(PPh_3)_4$ (11 mg,

9.5 μmol, 0.03 eq) in toluene (6 mL) and DMF (1.5 mL) was stirred overnight at 90 °C. After cooling to room temperature, the precipitate was collected by filtration and rinsed with hexanes. Further purification by silica gel chromatography column (hexanes/$CHCl_3$ = 4:1 to 2:1) gave the title product **BAI-2Tx** (210 mg, 77%) as a blue solid. ^{1}H NMR ($CDCl_3$, 500 MHz, 298 K): δ = 8.61 (d, *J* = 8.1 Hz, 2H), 8.33 (d, *J* = 8.0 Hz, 2H), 7.73 (d, *J* = 3.8 Hz, 2H), 7.60 (t, *J* = 7.5 Hz, 2H), 7.36 (t, *J* = 8.0 Hz, 2H), 7.28 (d, *J* = 3.5 Hz, 2H), 7.20 (d, *J* = 3.5 Hz, 2H), 6.75 (d, *J* = 3.5 Hz, 2H), 2.80 (d, *J* = 6.7 Hz, 4H), 1.65 (m, 2H), 1.27-1.41 (m, 16H), 0.96 (m, 12H). ^{13}C NMR ($CDCl_3$, 125 MHz, 298 K): δ =158.64, 145.19, 143.66, 143.31, 134.80, 133.18, 131.85, 131.20, 128.79, 126.29, 125.93, 124.84, 124.42, 124.17, 122.25, 121.83, 117.84, 41.47, 34.24, 32.41, 29.74, 28.92, 25.55, 23.04, 14.20, 10.90. MS (MALDI-TOF) *m/z* (%): 862.41 (100) [M^+]. HRMS (MALDI-TOF) [M^+] calculated: 862.2755, found: 862.4066.

BAI-3Tx: Under N_2 atmosphere, a deoxygenated mixture of **1** (200 mg, 0.320 mmol, 1.0 eq.), (5'-(2-decyltetradecyl)-[2,2'-bithiophen]-5-yl)trimethylstannane (484 mg, 0.730 mmol, 2.3 eq.), and $Pd(PPh_3)_4$ (18.0 mg, 16.0 μmol, 0.05 eq.) in toluene (12 mL) and DMF (3 mL) was stirred overnight at 90 °C. After cooling to room temperature, the precipitate was collected by filtration and rinsed with Hexanes. Further purification by silica gel chromatography column (Hexanes/$CHCl_3$ = 4:1 to 2:1) gave the title product **BAI-3Tx** (383 mg, 82%) as a green solid. ^{1}H NMR ($CDCl_3$, 500 MHz, 328 K): δ = 8.51 (d, *J* = 8.2 Hz, 2H), 8.30 (d, *J* = 8.0 Hz, 2H), 7.71 (d, *J* = 3.9 Hz, 2H), 7.53 (t, *J* = 8.0 Hz, 2H), 7.33 (t, *J* = 7.80 Hz, 2H), 7.26 (d, *J* = 4.0 Hz, 2H), 7.21 (d, *J* = 3.9 Hz, 2H), 7.03 (d, *J* = 3.8 Hz, 2H), 7.01 (d, J = 3.6 Hz, 2H), 6.68 (d, *J* = 3.6 Hz, 2H), 2.76 (d, *J* = 6.5 Hz, 4H), 1.66 (m, 2H), 1.27-1.291 (m, 80H), 0.89-0.92 (m, 12H). ^{13}C NMR ($CDCl_3$, 125 MHz, 328 K): δ = 158.39, 144.52, 143.46, 142.60, 137.87, 135.16, 134.63, 133.80, 131.77, 131.28, 128.35, 126.26, 126.04, 125.80, 125.07, 124.81, 124.16, 123.80, 123.55, 122.66, 121.69, 120.36, 117.74, 39.98, 34.62, 33.17, 31.97, 30.00, 29.71, 29.41, 26.62, 22.73, 14.17. MS (MALDI-TOF) *m/z* (%): 1474.89 (100) [M^+]. HRMS (MALDI-TOF) [M^+] calculated: 1474.7518, found: 1474.8524.

3: To a refluxing xylene (50 mL) suspension of 6,6'-dibromo-indigo **2** (0.500 g, 1.19 mmol, 1.0 eq.), a solution of 2-thiopheneacetyl chloride (0.650 mL, 5.24 mmol, 4.4 eq.) in xylene (5 mL) was added dropwise over 30 minutes. The half and double annulated indigo derivatives were observed as purple and red spots on the thin layer chromatography (TLC) plate respectively. The reaction was stopped when the purple spot completely disappeared as monitored by TLC. After cooling to room temperature, the precipitate was filtered and rinsed with THF, yielding the title product **3** (368 mg, 49%) as a red solid. ^{1}H NMR ($CDCl_3$, 500 MHz, 328 K): δ = 8.40 (d, J = 1.7 Hz, 2H), 8.08 (d, J = 8.5 Hz, 2H), 7.75-7.76 (m, 4H), 7.49 (dd, J = 5.4 Hz, J = 1.8 Hz, 2H), 7.32 (dd, J = 5.0 Hz, J = 3.7 Hz, 2H). Carbon NMR was not obtained due to limited solubility in common solvents. MS (MALDI-TOF) m/z (%): 629.93 (100) [M^+]. HRMS (MALDI-TOF) [M^+] calculated: 629.8707, found: 629.9200.

BAI-1T$_x$-1T$_y$: Synthesis follows the procedure of **BAI-2T$_x$**. Yield: 80%. ^{1}H NMR ($CDCl_3$, 500 MHz, 298 K): δ = 8.79 (s, 2H), 8.13 (d, J = 8.2 Hz, 2H), 7.85 (s, 2H), 7.75 (d, J = 5.2 Hz, 2H), 7.48 (d, J = 7.8 Hz, 2H), 7.34-7.37 (m, 4H), 6.77 (d, J = 3.4 Hz, 2H), 2.76 (d, J = 7.0 Hz, 4H), 1.67 (m, 2H), 1.29-1.32 (m, 48H), 0.90-0.92 (m, 12H). ^{13}C NMR ($CDCl_3$, 125 MHz, 328 K): δ =158.81, 144.48, 140.38, 139.46, 136.41, 134.86, 130.21, 130.17, 130.08, 126.96, 126.50, 125.07, 124.94, 124.31, 124.23, 122.27, 118.20, 31.92, 30.92, 29.71, 29.67, 29.63, 29.48, 29.43, 29.37, 28.98, 22.69, 14.13. MS (MALDI-TOF) m/z (%): 1086.65 (100) [M^+]. HRMS (MALDI-TOF) [M^+] calculated: 1086.5259, found: 1086.6461.

BAI-1T$_x$-2T$_y$: Synthesis follows the procedure of **BAI-2T$_x$**. Yield: 73%. ^{1}H NMR ($CDCl_3$, 500 MHz): δ = 8.70 (s, 2H), 8.08 (d, J = 8.3 Hz, 2H), 7.84 (d, J = 3.5 Hz, 2H), 7.74 (dd, J = 5.1 Hz, J = 1.1 Hz, 2H), 7.40 (dd, J = 8.4 Hz, J = 1.6 Hz, 2H), 7.34 (dd, J = 5.1 Hz, J = 3.7 Hz, 4H), 7.03 (d, J = 3.8 Hz, 2H), 7.00 (d, J = 3.4 Hz, 2H), 6.65 (d, J = 3.5 Hz, 2H), 2.73 (d, J = 6.5 Hz, 4H), 1.65 (m, 2H), 1.31-1.33 (m, 80H), 0.92-0.93 (m, 12H). ^{13}C NMR ($CDCl_3$, 125 MHz, 328 K): δ = 158.21, 137.77 135.21, 134.76, 134.72, 130.18, 130.00, 129.84, 127.11, 127.10, 126.95, 126.78, 126.76, 126.19, 125.33, 125.01, 124.88, 122.24, 118.17, 118.08, 110.82, 37.86, 35.53, 31.95, 31.29, 30.07, 29.73, 29.69, 29.65, 29.40, 29.38, 26.85, 22.72, 14.16. MS (MALDI-TOF) m/z (%): 1474.98 (100) [M^+]. HRMS (MALDI-TOF) [M^+] calculated: 1474.7518, found: 1474.8894.

4: NBS (34.0 mg, 0.190 mmol, 2.1 eq) was added to an ice-bath cooled solution of **BAI-1T$_x$-1T$_y$** (100 mg, 91.0 μmol, 1.0 eq.) in $CHCl_3$. The reaction was allowed to warm up to room temperature and then refluxed overnight. The reaction mixture was partitioned with water and dried with brine and $MgSO_4$. The residue from filtration and vacuum evaporation was subjected to silica gel chromatography column with $CHCl_3$/Hexanes (1:1) as the eluent. The title product **4** (92.0 mg, 81%) was obtained as a red solid. ^{1}H NMR ($CDCl_3$, 500 MHz, 328 K): δ = 8.62 (s, 2H), 8.13 (d, J = 8.2 Hz, 2H), 7.70 (d, J = 3.5 Hz, 2H), 7.43 (d, J = 8.4 Hz, 2H), 7.31(m, 4H), 6.72 (s, 2H), 2.69 (d, J = 5.8 Hz, 4H), 1.64 (m, 2H), 1.28-1.29 (m, 48H), 0.90-0.92 (m, 12H). ^{13}C NMR ($CDCl_3$, 125 MHz, 328 K): δ = 158.57, 143.81, 140.93, 140.28, 134.91, 132.95, 132.12, 132.06, 130.71, 129.41, 126.32, 125.74, 125.30, 125.00, 124.31, 121.93, 117.87, 111.30, 31.91, 30.48, 29.69, 29.67, 29.65, 29.60, 29.49, 29.47, 29.36, 22.68, 14.12. MS (MALDI-TOF) *m/z* (%): 1242.44 (100) [M$^+$]. HRMS (MALDI-TOF) [M$^+$] calculated: 1242.3549, found: 1242.4368.

5: Synthesis follows the procedure of **4**. Yield: 77%. ^{1}H NMR ($CDCl_3$, 500 MHz, 328 K): δ = 8.35 (s, 2H), 8.03 (d, J = 8.3 Hz, 2H), 7.71 (s, 2H), 7.32 (d, J = 3.8 Hz, 2H), 7.23-7.25 (m, 2H), 7.13 (s, 2H), 6.88 (s, 4H), 6.58 (d, J = 3.1 Hz, 2H), 2.73 (d, J = 6.5 Hz, 4H), 1.65 (m, 2H), 1.31-1.33 (m, 80H), 0.92-0.93 (m, 12H). ^{13}C NMR ($CDCl_3$, 125 MHz, 328 K): δ = 158.42, 144.52, 143.46, 142.60, 137.88, 135.16, 134.63, 133.80, 131.78, 131.28, 128.36, 126.26, 126.04, 125.80, 125.07, 124.82, 124.16, 123.80, 123.56, 122.67, 121.69, 117.73, 39.98, 34.62, 33.17, 31.97, 30.00, 29.71, 29.41, 26.62, 22.73, 14.17. MS (MALDI-TOF) *m/z* (%): 1630.75 (100) [M$^+$]. HRMS (MALDI-TOF) [M$^+$] calculated: 1630.5728, found: 1630.7416.

BAI-2T$_x$-1T$_y$: Synthesis follows the procedure of **BAI-2T$_x$**. Yield: 56%. ^{1}H NMR ($C_2D_2Cl_4$, 500 MHz, 353 K): δ = 8.74 (s, 2H), 8.23 (d, J = 8.4 Hz, 2H), 7.81 (d, J = 3.8 Hz, 2H), 7.50 (d, J = 8.5 Hz, 2H), 7.36 (d, J = 3.7 Hz, 4H), 7.25 (d, J = 3.5 Hz, 2H), 6.80 (m, 4H), 2.85 (d, J = 6.6 Hz, 4H), 2.80 (d, J = 6.4 Hz, 4H), 1.71 (m, 4H), 1.32-1.41 (m, 62H), 0.96-1.02 (m, 24H). ^{13}C NMR ($C_2D_2Cl_4$, 125 MHz, 353 K): δ = 158.37, 146.80, 145.12, 144.56, 142.57, 140.08, 138.44, 134.67, 133.49, 130.95, 128.89, 126.48, 126.03, 125.10, 124.76, 124.09, 123.69, 123.00, 122.59, 122.37, 122.04, 113.53, 41.40, 39.91, 34.83,

33.34, 32.54, 31.68, 31.65, 29.76, 29.46, 29.39, 29.38, 29.06, 28.84, 26.52, 26.49, 25.74, 22.79, 22.43, 13.85, 10.78. MS (MALDI-TOF) *m/z* (%): 1474.96 (100) [M$^+$]. HRMS (MALDI-TOF) [M$^+$] calculated: 1474.7518, found: 1474.9602.

BAI-2T$_x$-2T$_y$: Synthesis follows the procedure of **BAI-2T$_x$**. Yield: 43%. ^{1}H NMR ($CDCl_3$, 500 MHz, 328 K): δ = 8.56 (s, 2H), 8.20 (d, *J* = 8.3 Hz, 2H), 7.94 (d, *J* = 3.8 Hz, 2H), 7.37 (d, *J* = 3.7 Hz, 2H), 7.32 (d, *J* = 8.6 Hz, 2H), 7.26 (t, *J* = 3.1 Hz, 2H), 7.23 (d, *J* = 3.8 Hz, 2H), 6.96 (d, *J* = 3.7 Hz, 2H), 6.93 (d, *J* = 3.3 Hz, 2H), 6.77 (d, *J* = 3.5 Hz, 2H), 6.61 (d, *J* = 3.3 Hz, 2H), 2.84 (d, *J* = 6.5 Hz, 4H), 2.71 (d, *J* = 6.5 Hz, 4H), 1.74-1.77 (m, 2H), 1.65 (m, 2H), 1.313-1.40 (m, 160H), 0.87-0.96 (m, 24H). ^{13}C NMR ($CDCl_3$, 125 MHz, 328 K): δ = 158.29, 144.94, 144.64, 144.35, 143.00, 141.80, 140.35, 139.26, 137.35, 135.15, 134.56, 133.79, 131.31, 128.38, 126.14, 125.85, 125.20, 124.12, 123.75, 123.22, 122.50, 121.90, 112.99, 40.15, 39.96, 34.95, 34.68, 33.44, 33.34, 31.87, 30.00, 29.94, 29.66, 29.61, 29.28, 26.73, 26.63, 22.59, 13.93. MS (MALDI-TOF) *m/z* (%): 2311.71 (100) [M$^+$]. HRMS (MALDI-TOF) [M$^+$] calculated: 2311.4784, found: 2311.7158.

Table S1. Characters of the lowest vertical singlet excitations for BAI-derivatives obtained from TD-DFT calculations, wavelength (λ in nm), oscillator strength (f), dominant transition and its total weight.

Molecule	λ, nm	f	from	to	weight, %
BAI-1Tx	586	0.77	HOMO	LUMO	100
	424	0.12	HOMO-2	LUMO	99
	336	0.03	HOMO-4	LUMO	74
	331	0.11	HOMO	LUMO+2	82
	296	0.58	HOMO-1	LUMO+1	89
BAI-2Tx	743	1.49	HOMO	LUMO	99
	483	0.05	HOMO-2	LUMO	96
	396	0.24	HOMO-4	LUMO	83
	380	0.30	HOMO	LUMO+2	86
	360	0.29	HOMO-1	LUMO+1	85
BAI-3Tx	817	1.93	HOMO	LUMO	100
	527	0.13	HOMO-2	LUMO	93
	441	0.79	HOMO	LUMO+2	87
	426	0.10	HOMO-4	LUMO	91
	409	0.42	HOMO-1	LUMO+1	93
BAI-1Tx-1Ty	597	0.68	HOMO	LUMO	98
	529	**0.73**	HOMO-2	LUMO	98
	417	0.02	HOMO-4	LUMO	93
	371	1.20	HOMO-1	LUMO+1	97
BAI-2Tx-1Ty	746	1.34	HOMO	LUMO	100
	537	**0.64**	HOMO-2	LUMO	98
	480	0.03	HOMO-4	LUMO	94
	401	0.90	HOMO-1	LUMO+1	88
	398	0.42	**HOMO**	**LUMO+2**	81

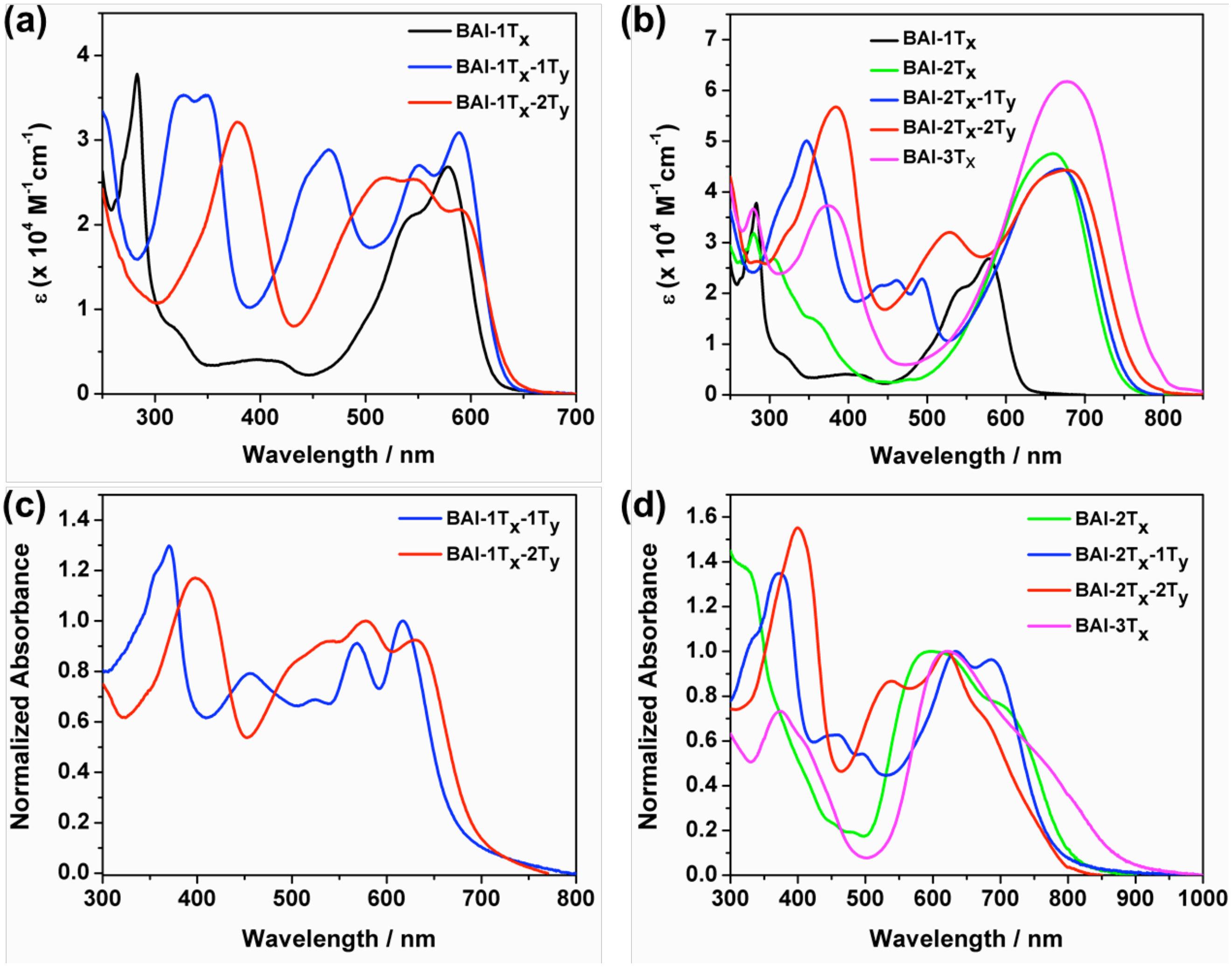

Figure S1. UV-vis absorption of the **BAIs** in solution (a, b, extinction coefficient vs. concentration) and in thin film (c,d, normalized).

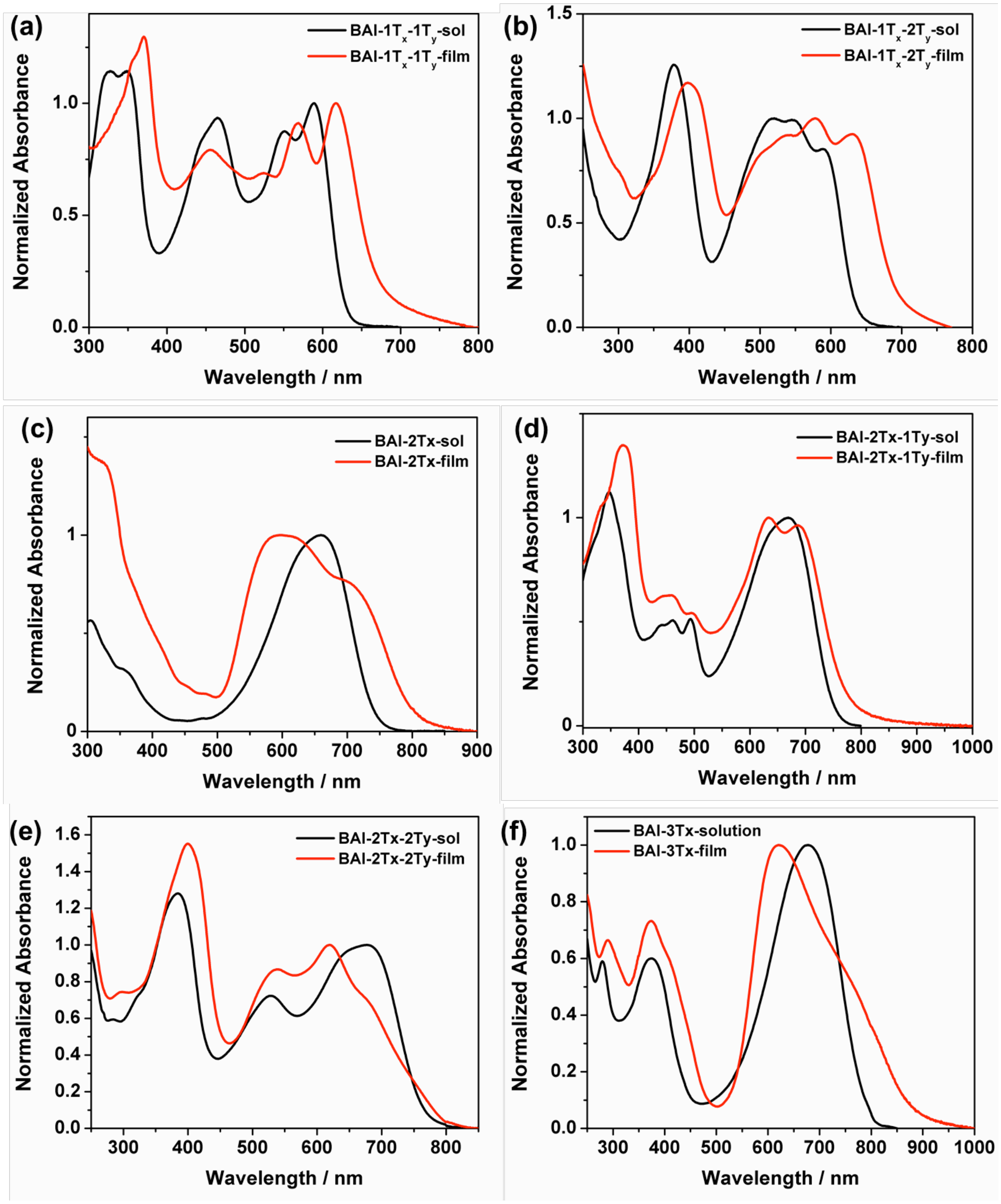

Figure S2. Comparison of UV-vis absorption of the individual **BAIs** (solution vs. thin film).

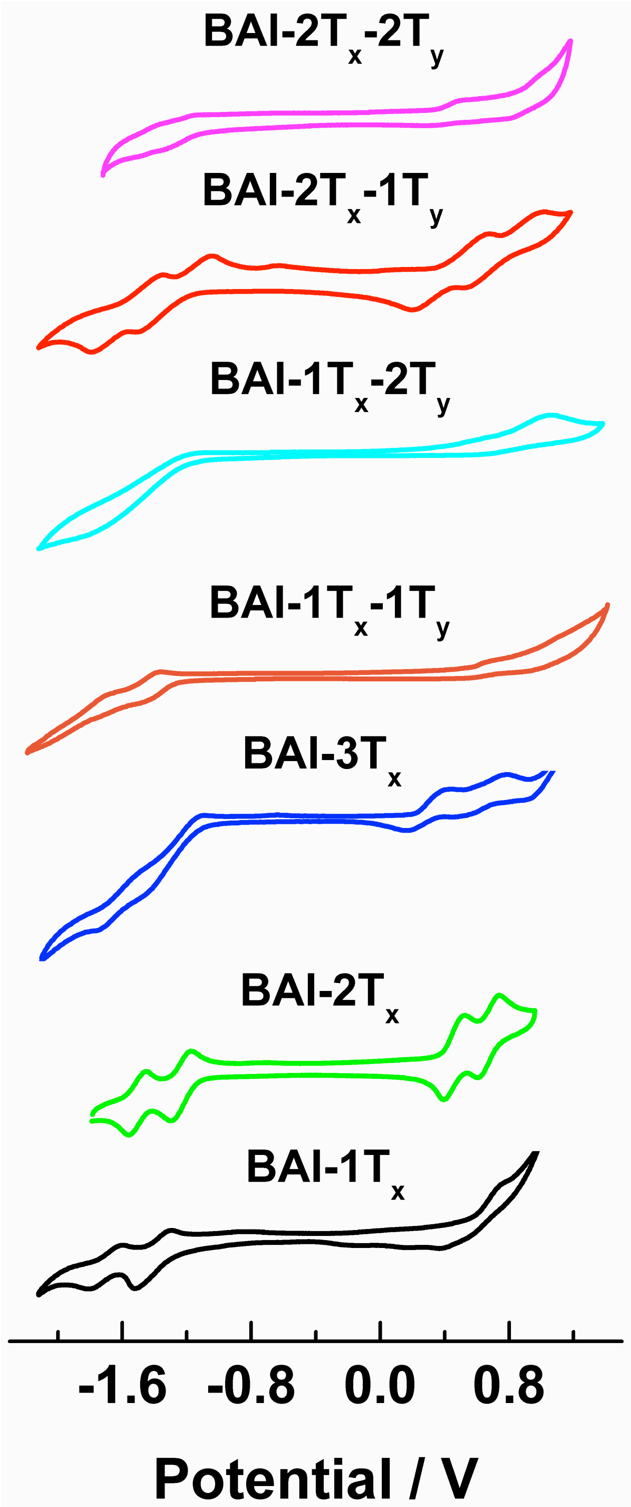

Figure S3. Cyclic voltammetry of the **BAI**s in $CHCl_3$ (vs. F_c/F_c^+).

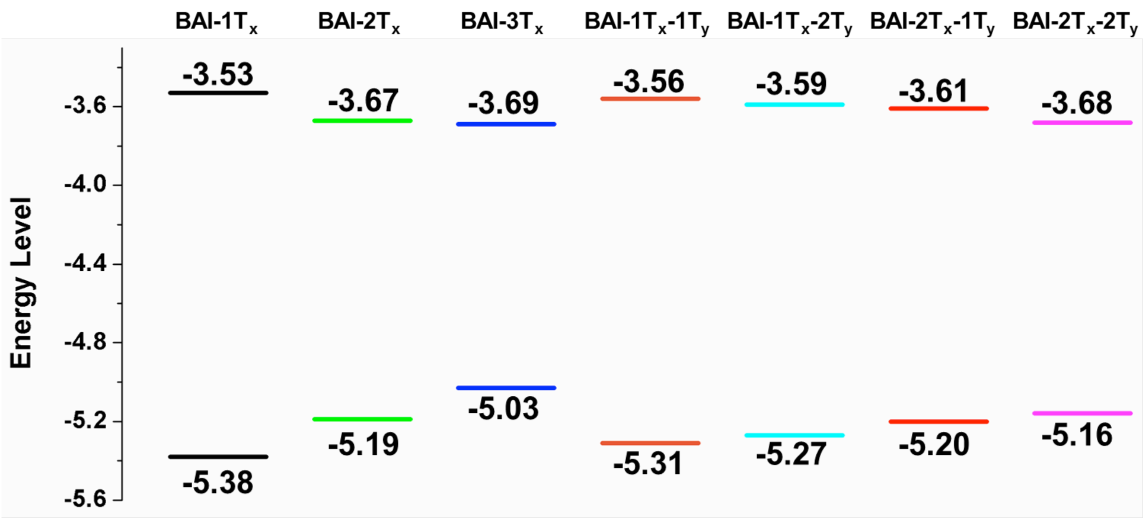

Figure S4. Experimental energy level diagram of the **BAI**s.

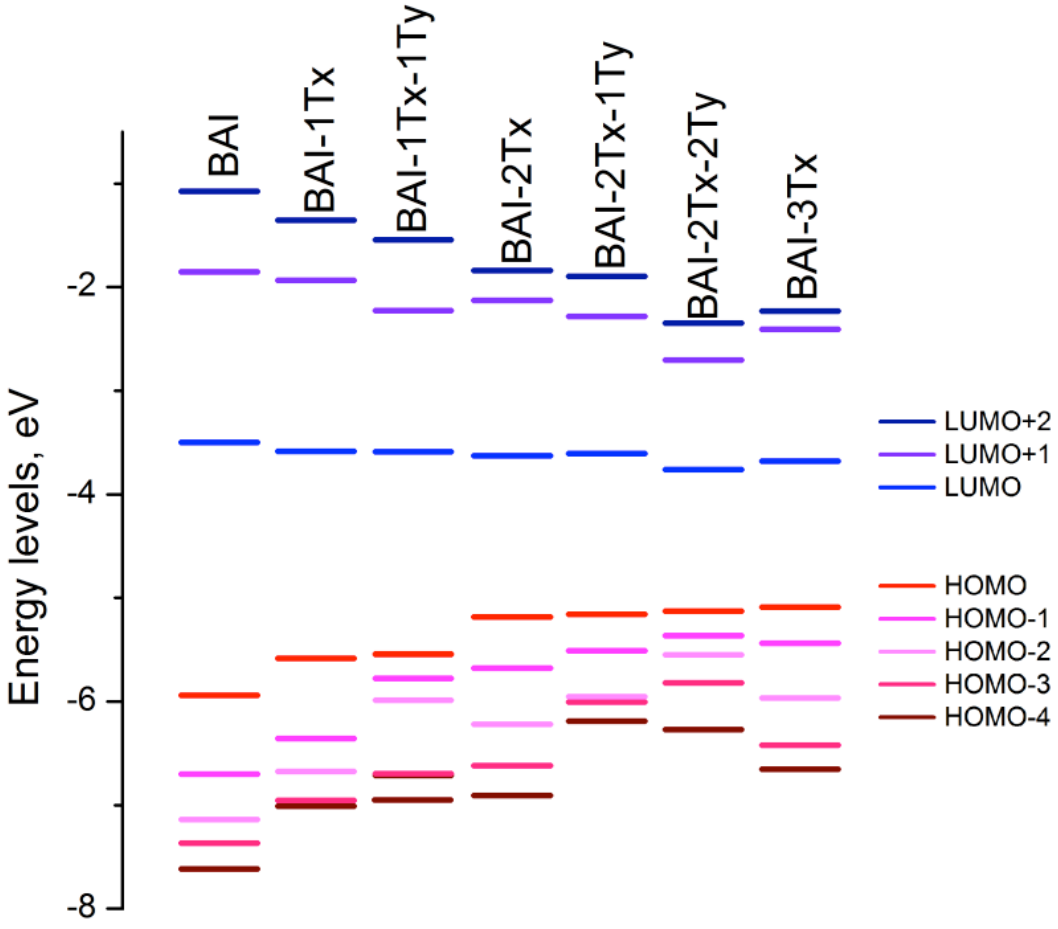

Figure S5. Calculated energy level diagram of the functionalized **BAI**s in chloroform.

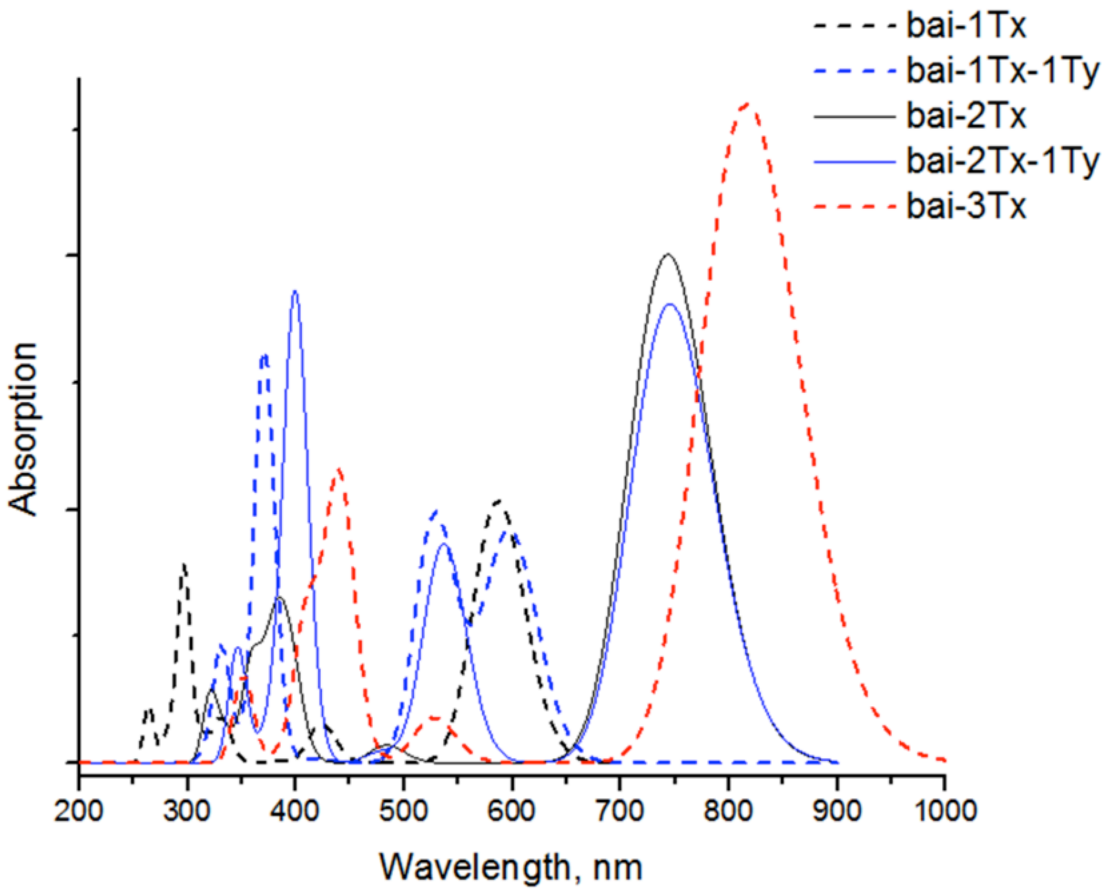

Figure S6. Simulated (TDDFT) steady-state absorption spectra of **BAI**s in chloroform. The vertical excitation energies with oscillator strength are listed in Table S1.

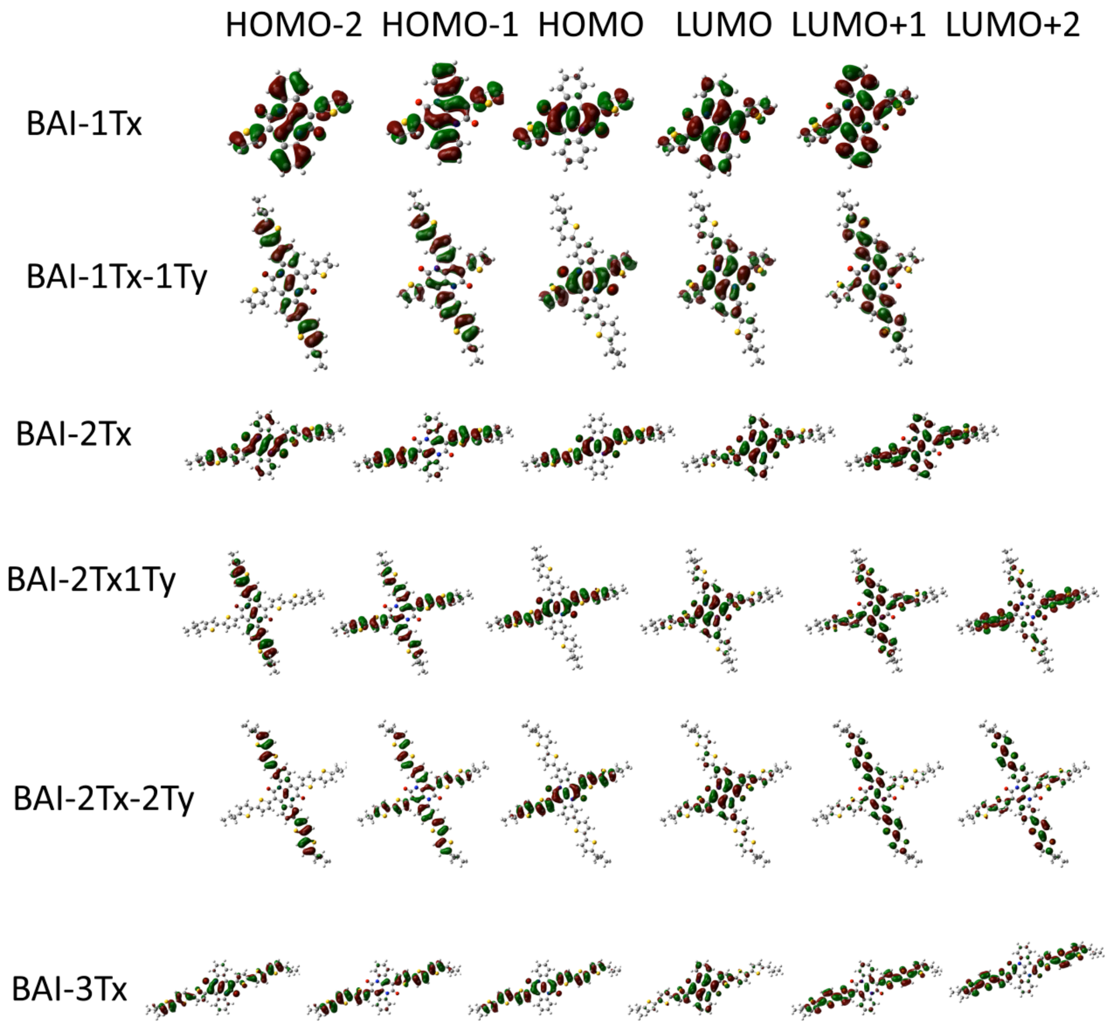

Figure S7. Deeper occupied and unoccupied frontier orbitals of the **BAI**s.

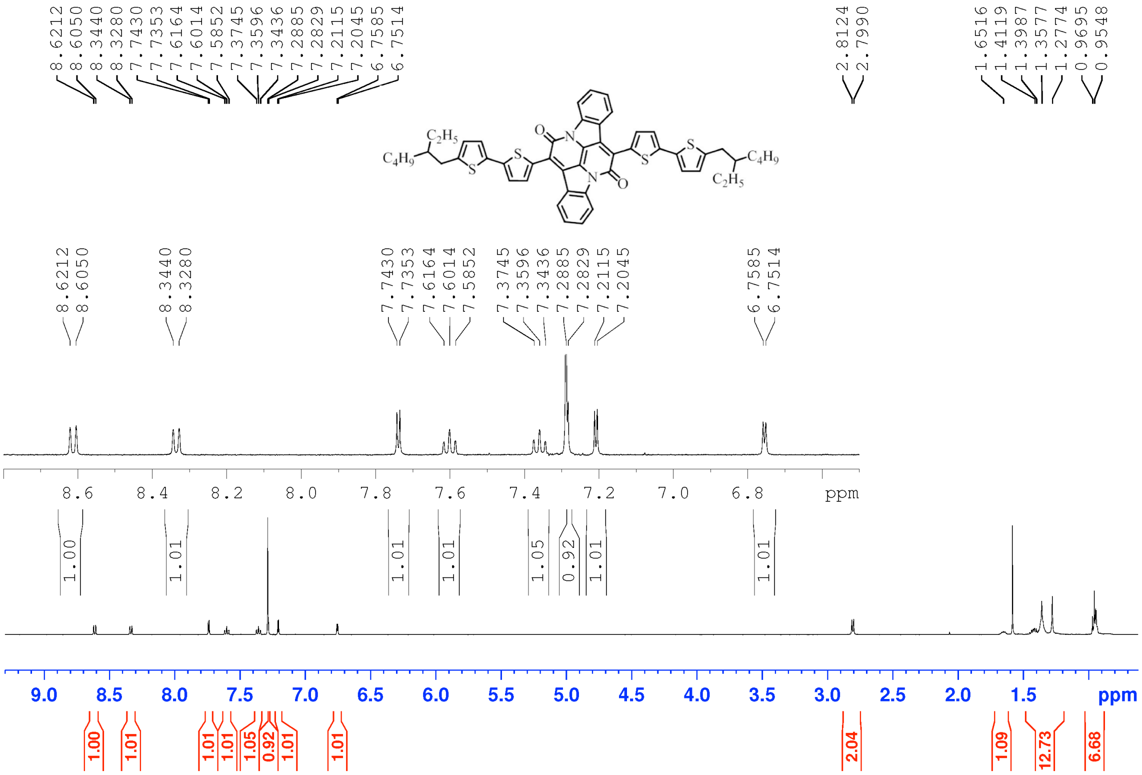

Figure S8. ^{1}H NMR spectrum of **BAI-2T$_x$** ($CDCl_3$, 298 K).

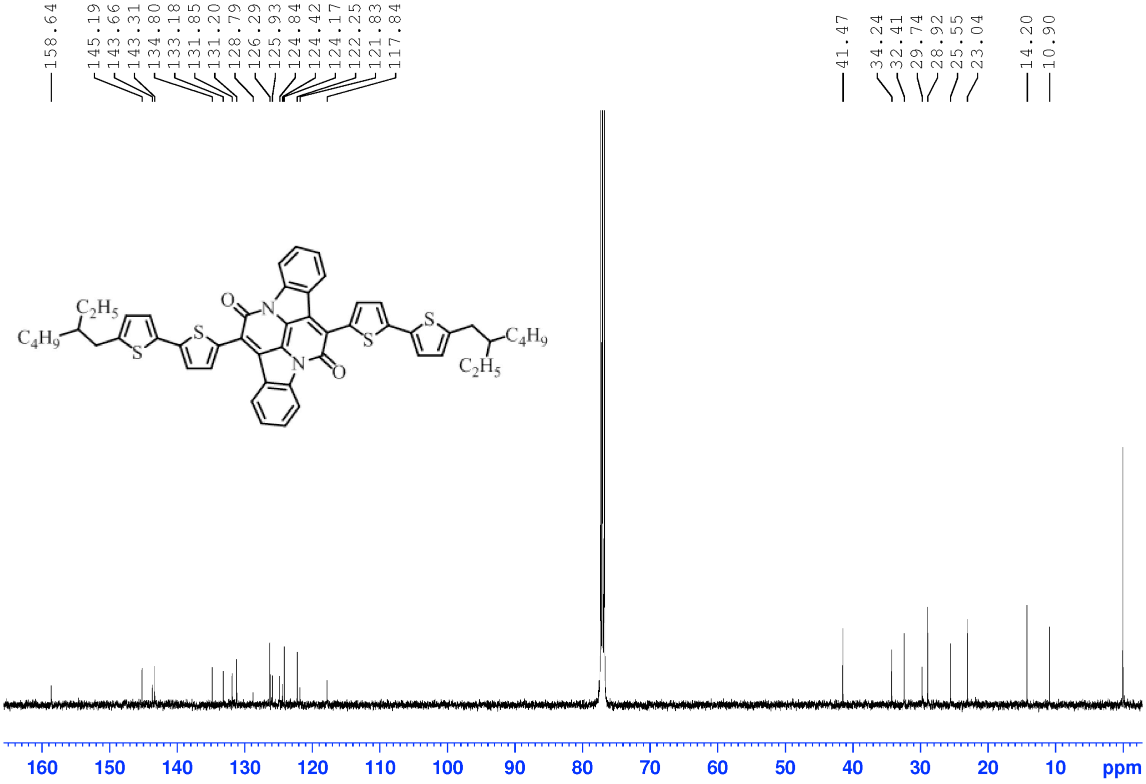

Figure S9. ^{13}C NMR spectrum of **BAI-2T$_x$** ($CDCl_3$, 298 K).

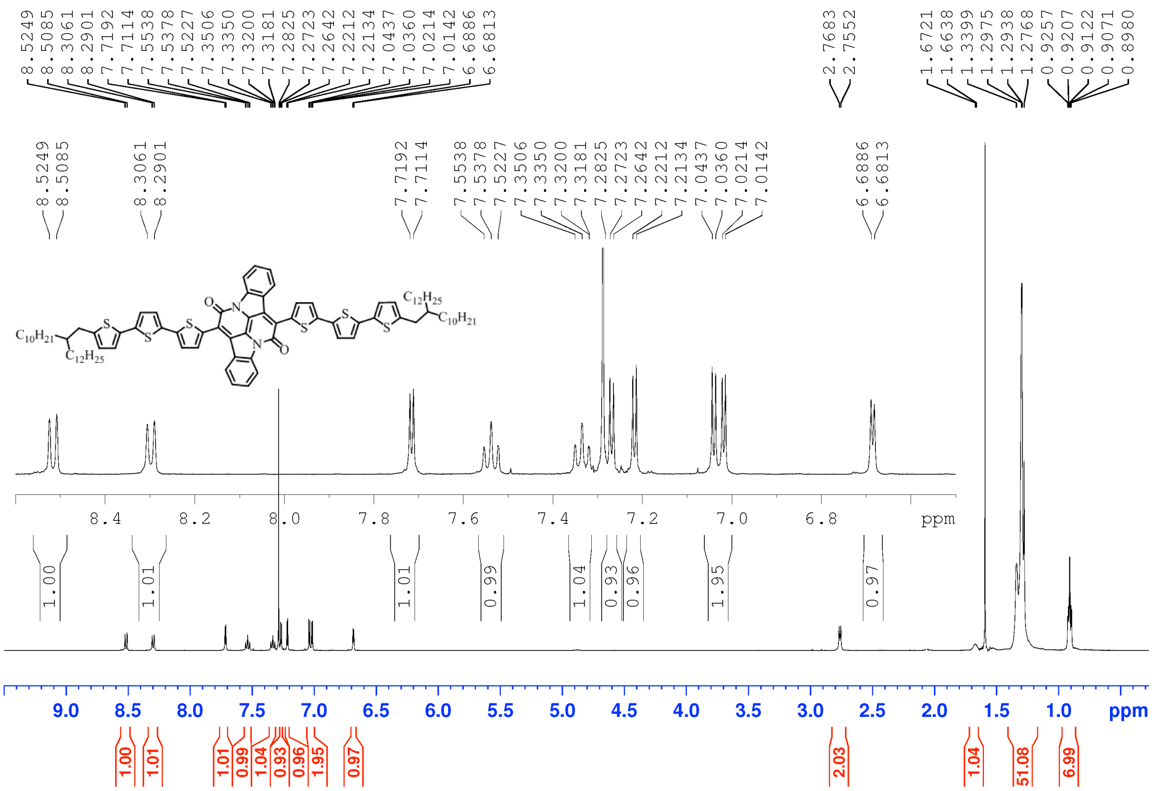

Figure S10. ^{1}H NMR spectrum of **BAI-3T$_{x}$** ($CDCl_3$, 328 K).

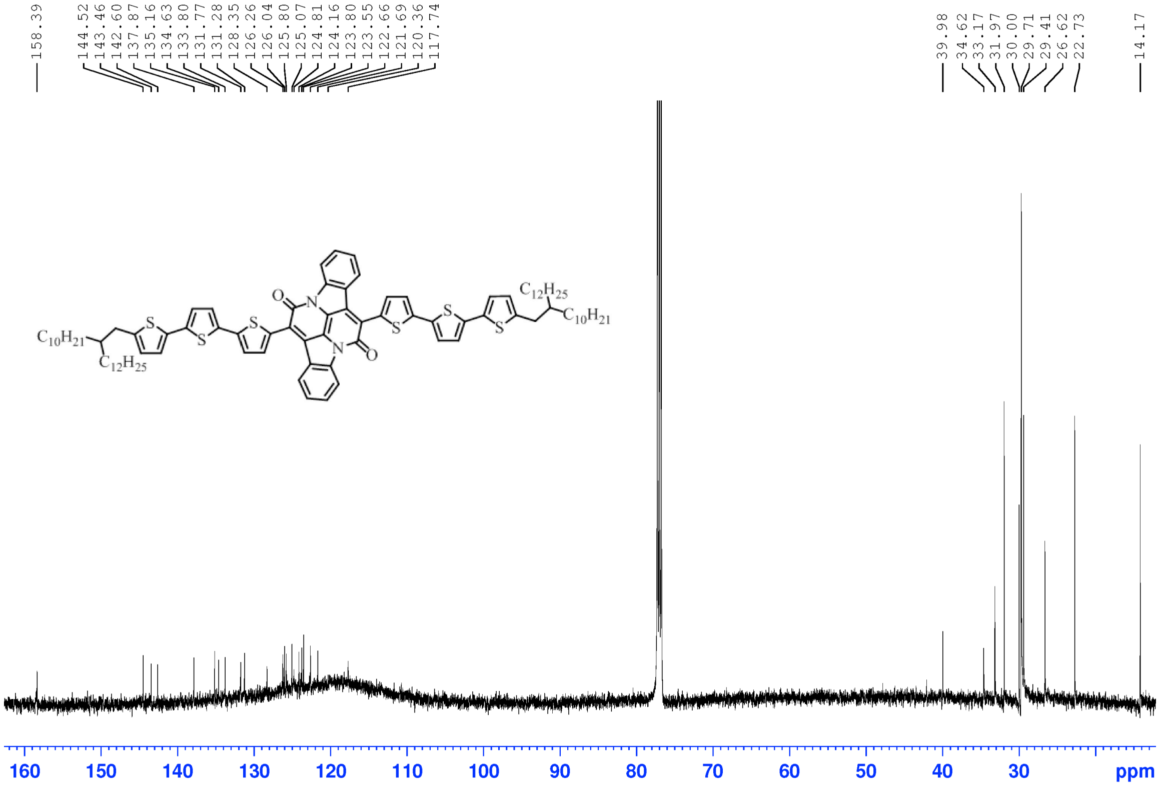

Figure S11. ^{13}C NMR spectrum of **BAI-3T$_{x}$** ($CDCl_3$, 328 K).

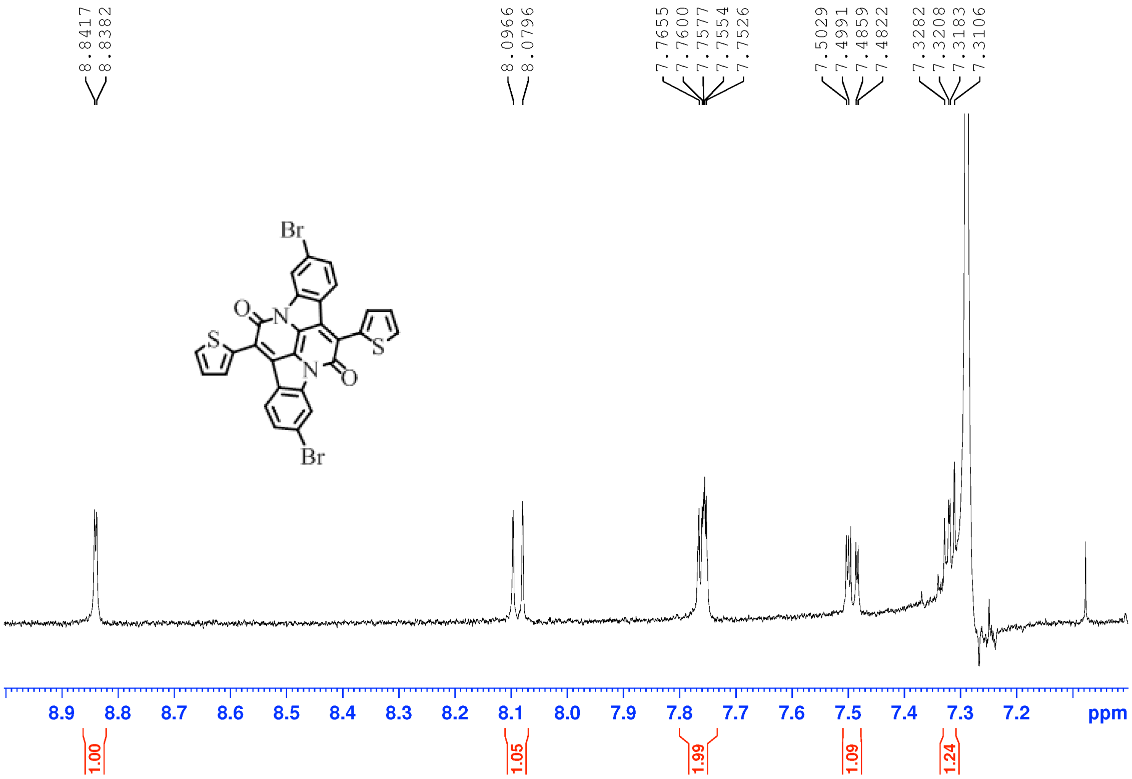

Figure S12. ^{1}H NMR spectrum of **3** ($CDCl_3$, 328 K).

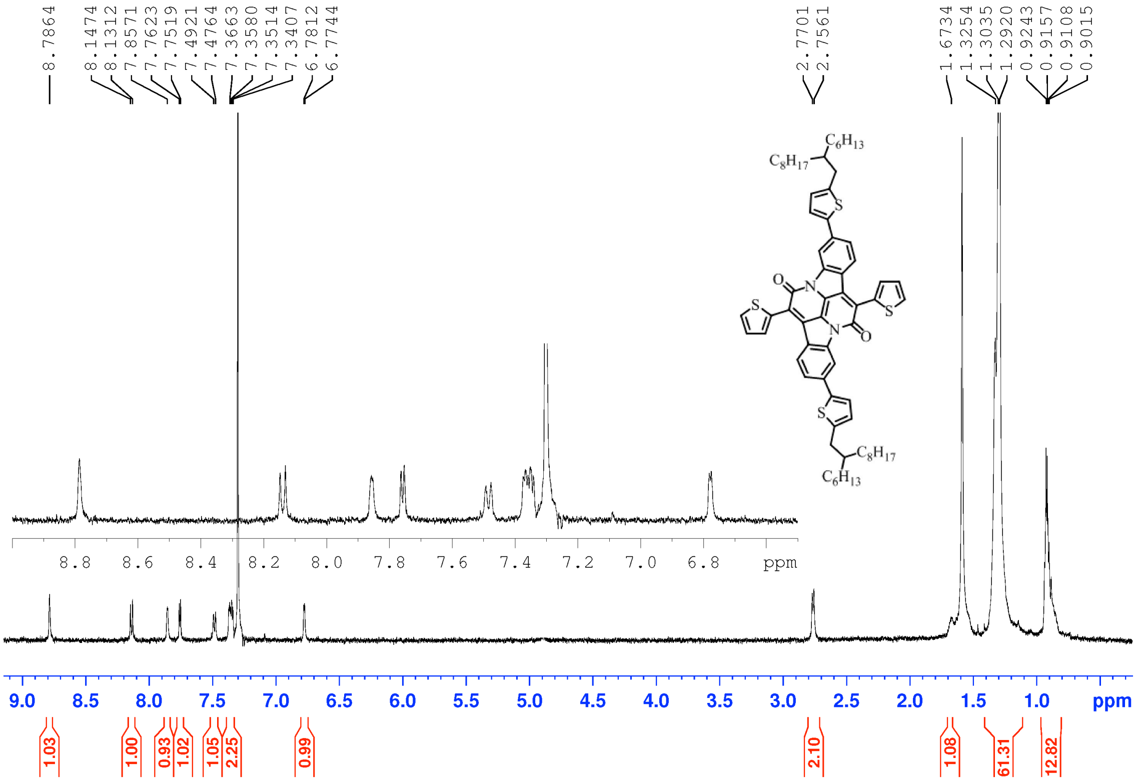

Figure S13. ^{1}H NMR spectrum of **BAI-1T$_x$-1T$_y$** ($CDCl_3$, 298 K).

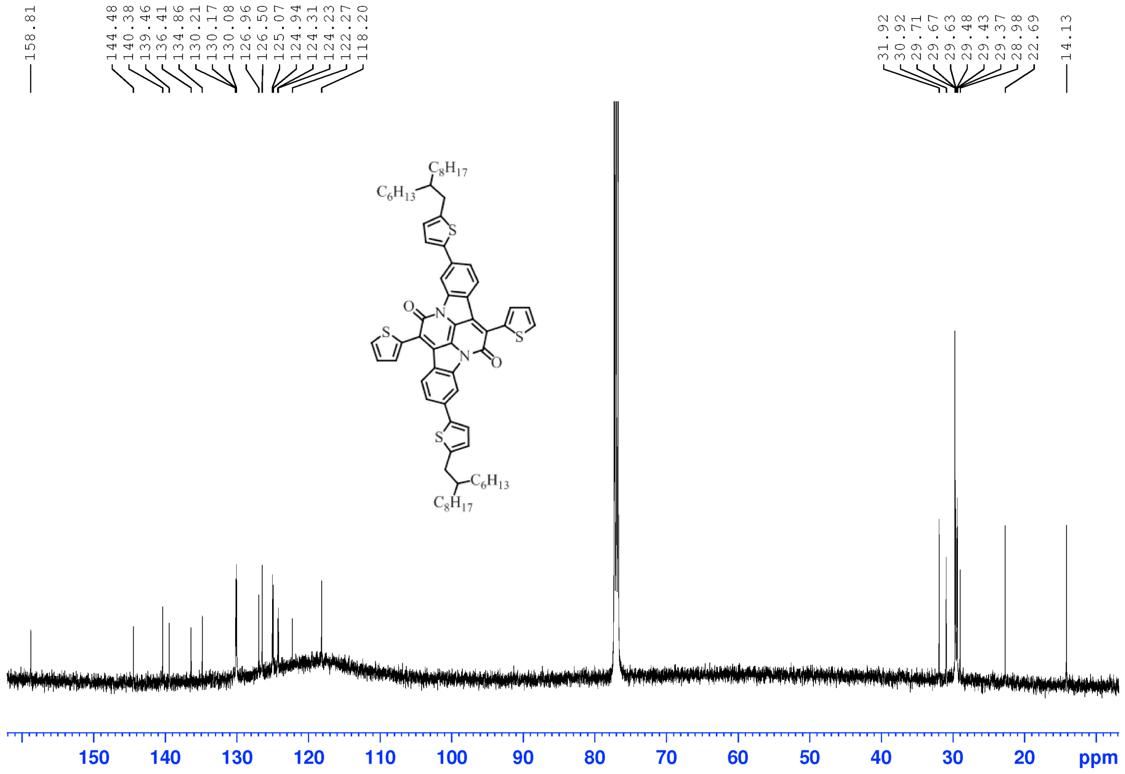

Figure S14. ^{13}C NMR spectrum of **BAI-1T$_x$-1T$_y$** ($CDCl_3$, 328 K).

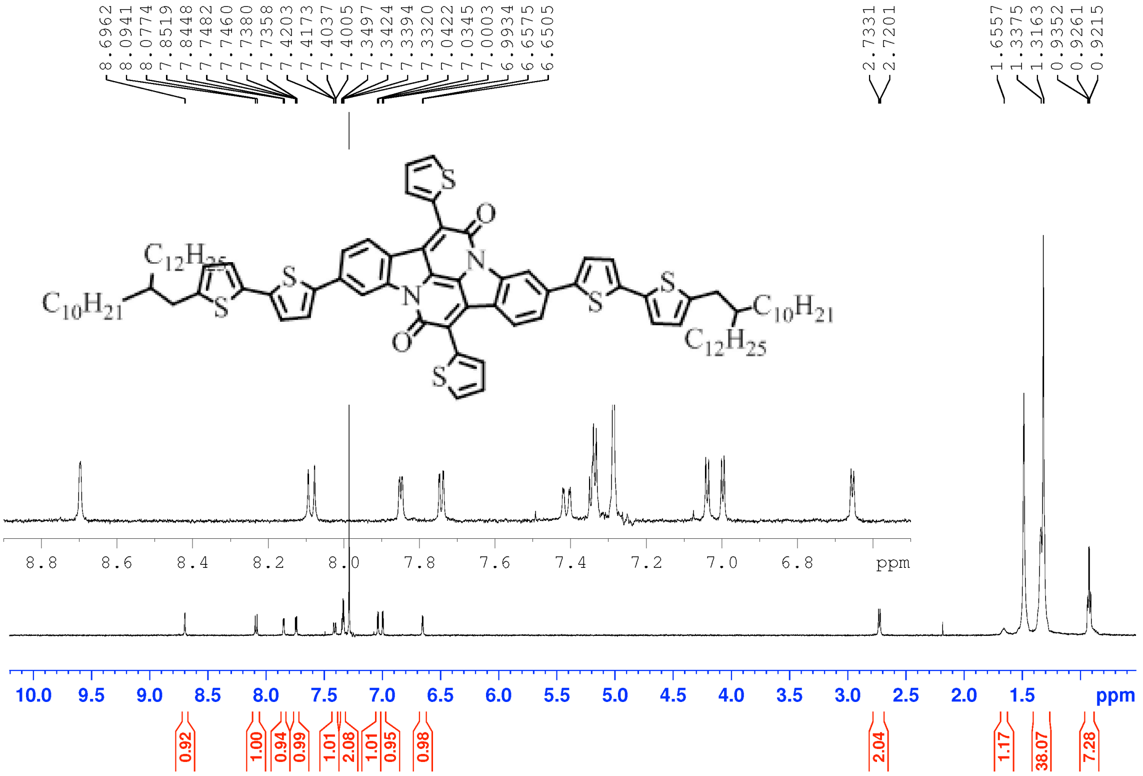

Figure S15. ^{1}H NMR spectrum of **BAI-1T$_x$-2T$_y$** ($CDCl_3$, 328 K).

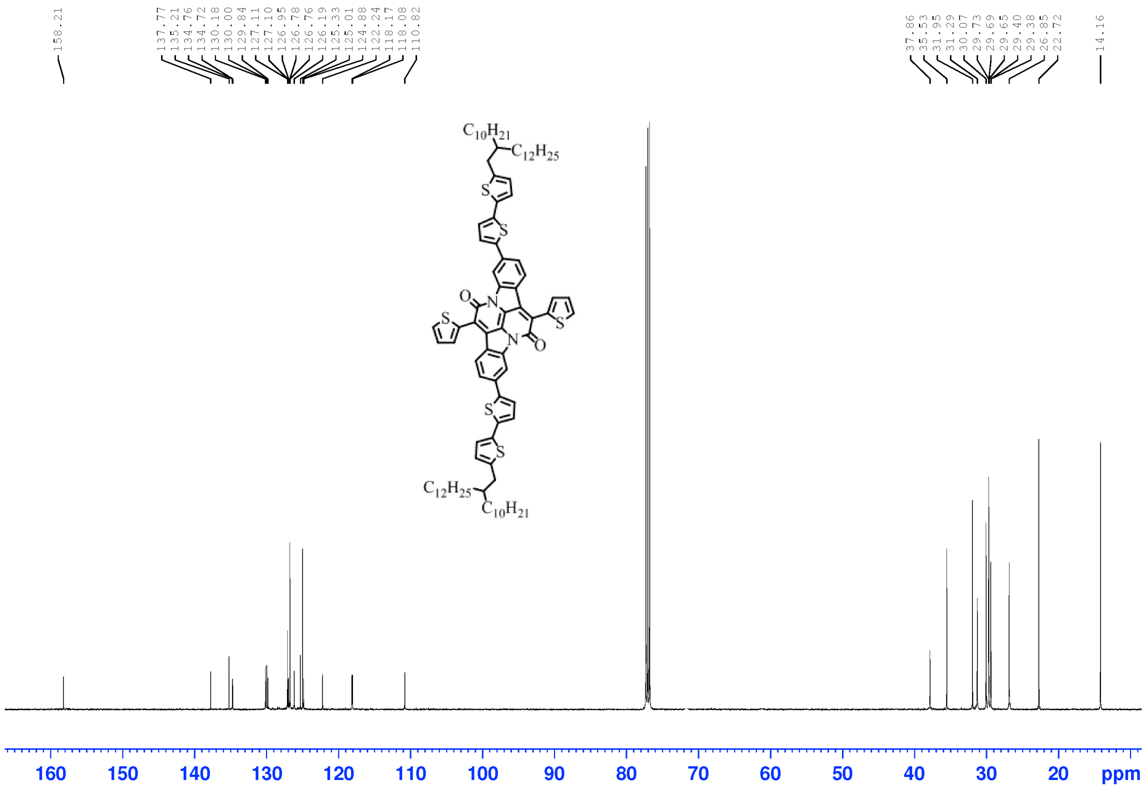

Figure S16. ^{13}C NMR spectrum of **BAI-1T$_x$-2T$_y$** ($CDCl_3$, 328 K).

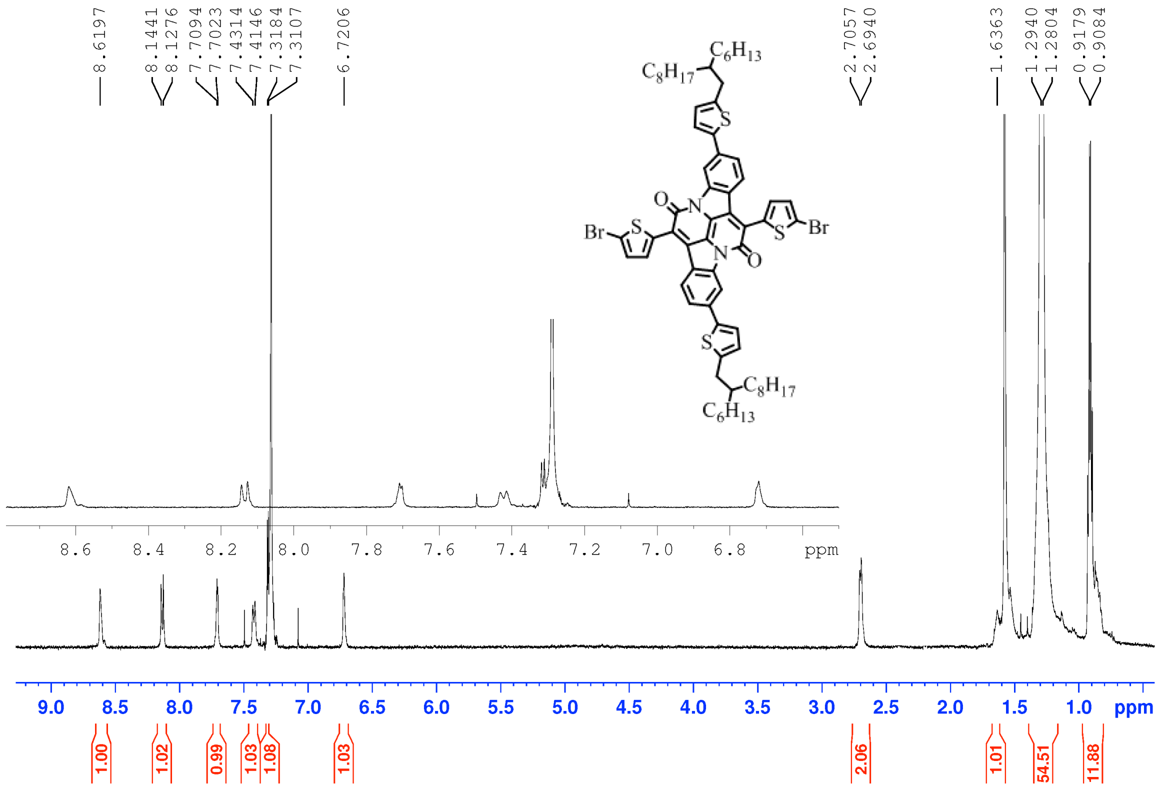

Figure S17. ^{1}H NMR spectrum of **4** ($CDCl_3$, 328 K).

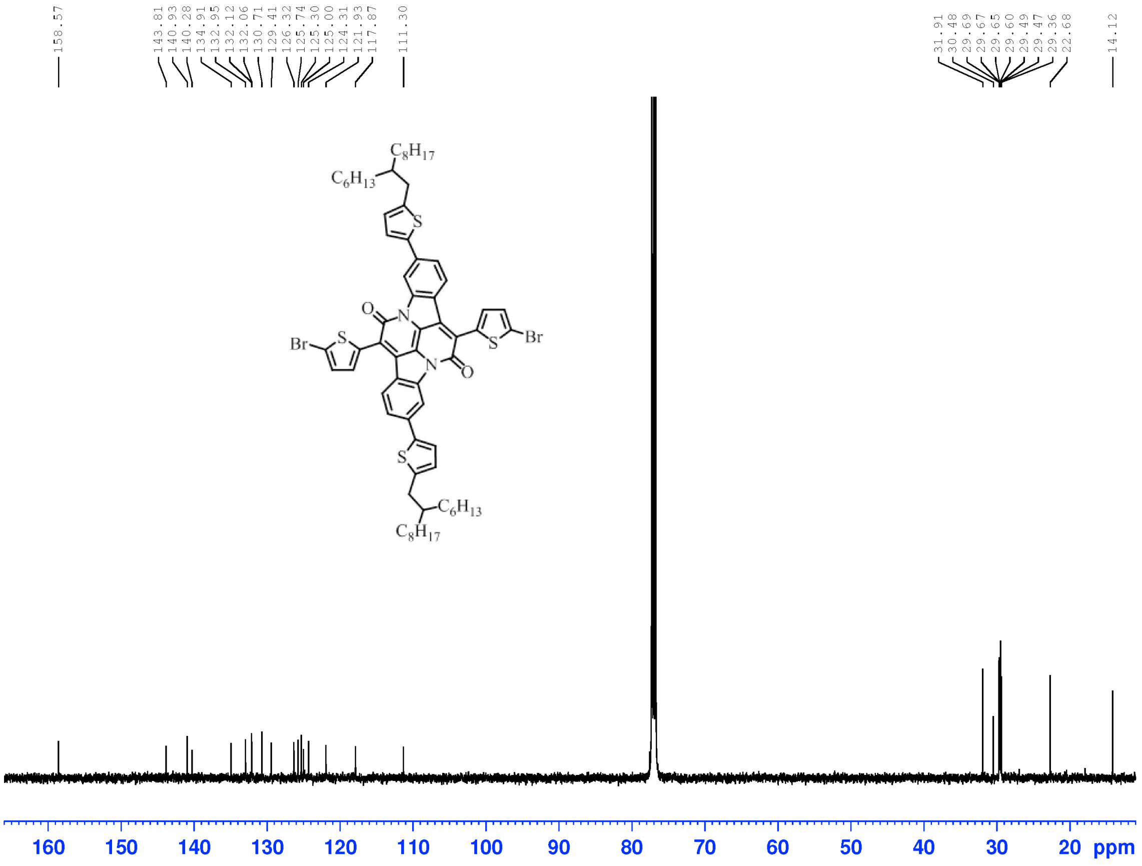

Figure S18. ^{13}C NMR spectrum of **4** ($CDCl_3$, 328 K).

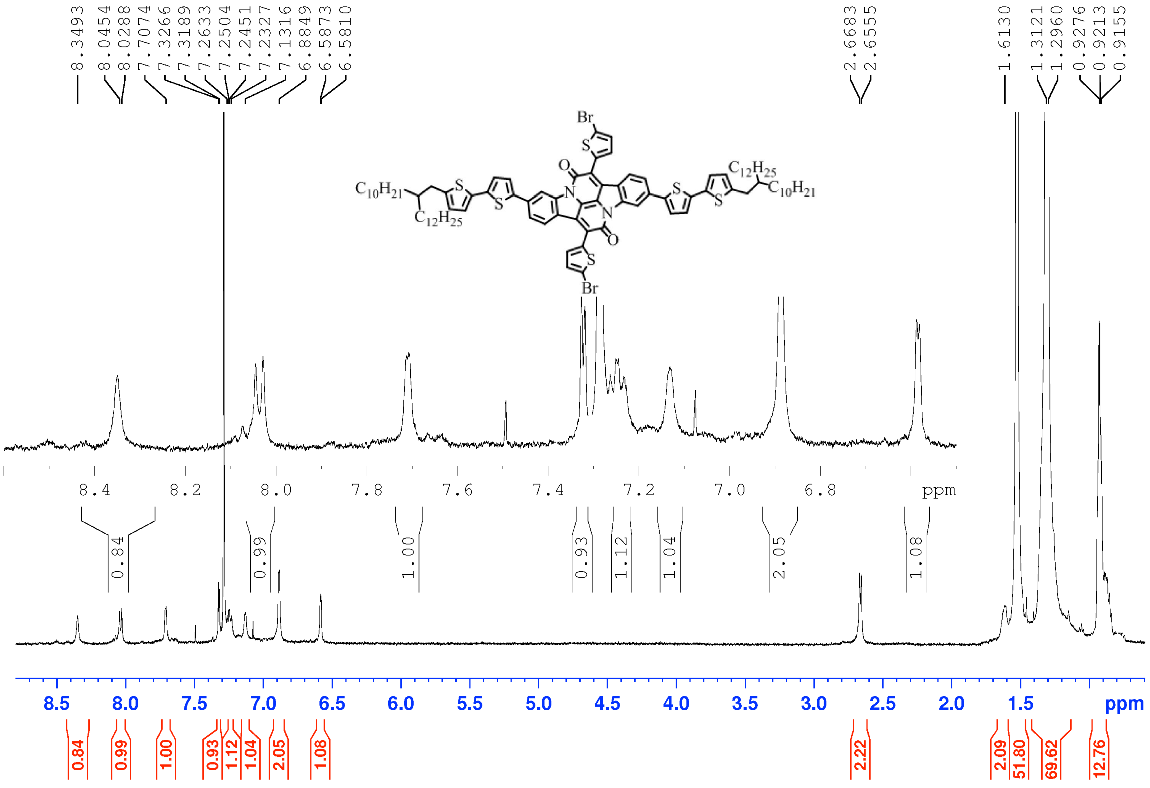

Figure S19. ^{1}H NMR spectrum of **5** ($CDCl_3$, 328 K).

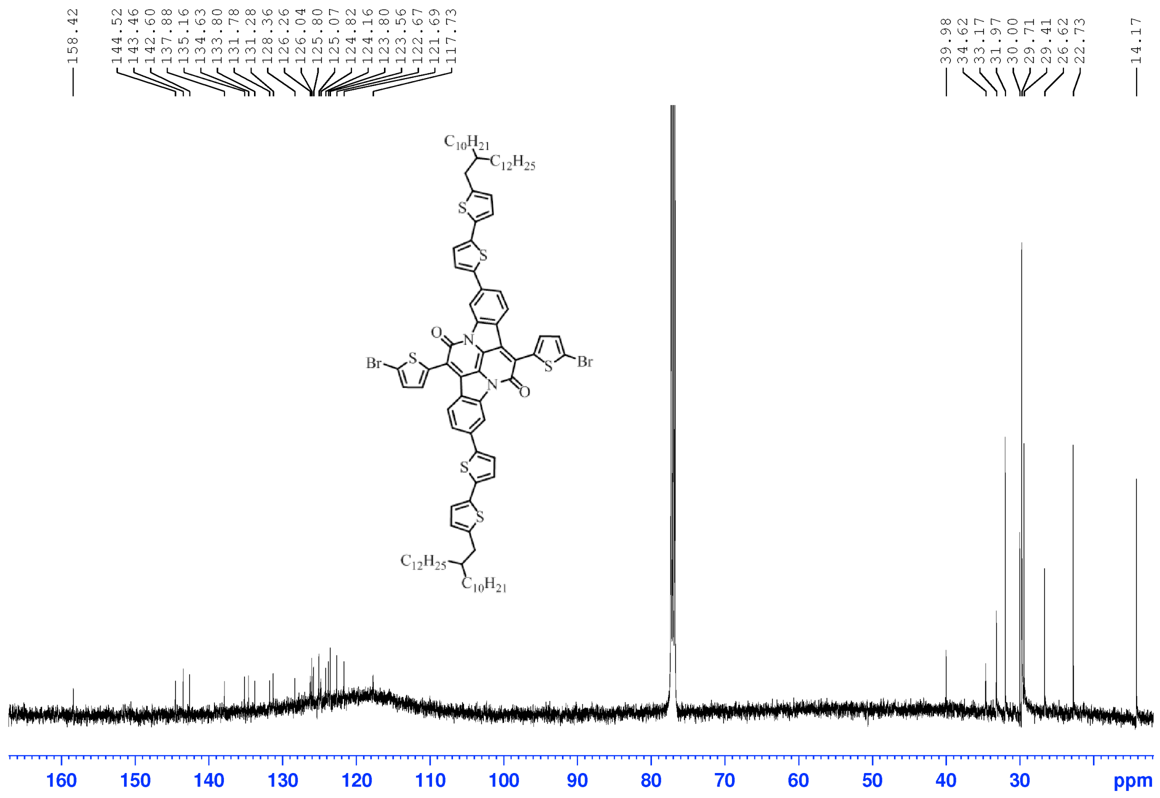

Figure S20. ^{13}C NMR spectrum of **5** ($CDCl_3$, 328 K).

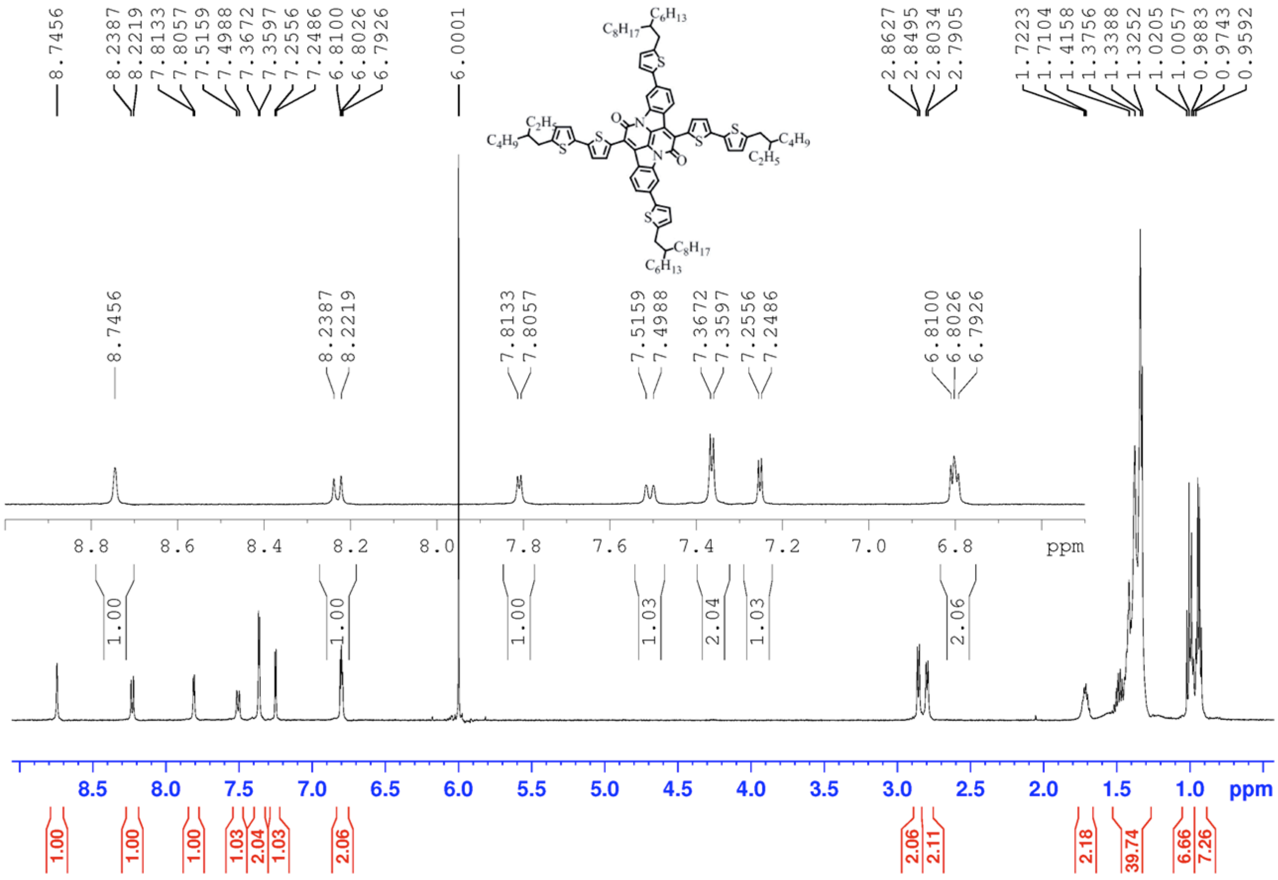

Figure S21. ^{1}H NMR spectrum of **BAI-2T$_x$-1T$_y$** ($C_2D_2Cl_4$, 353 K).

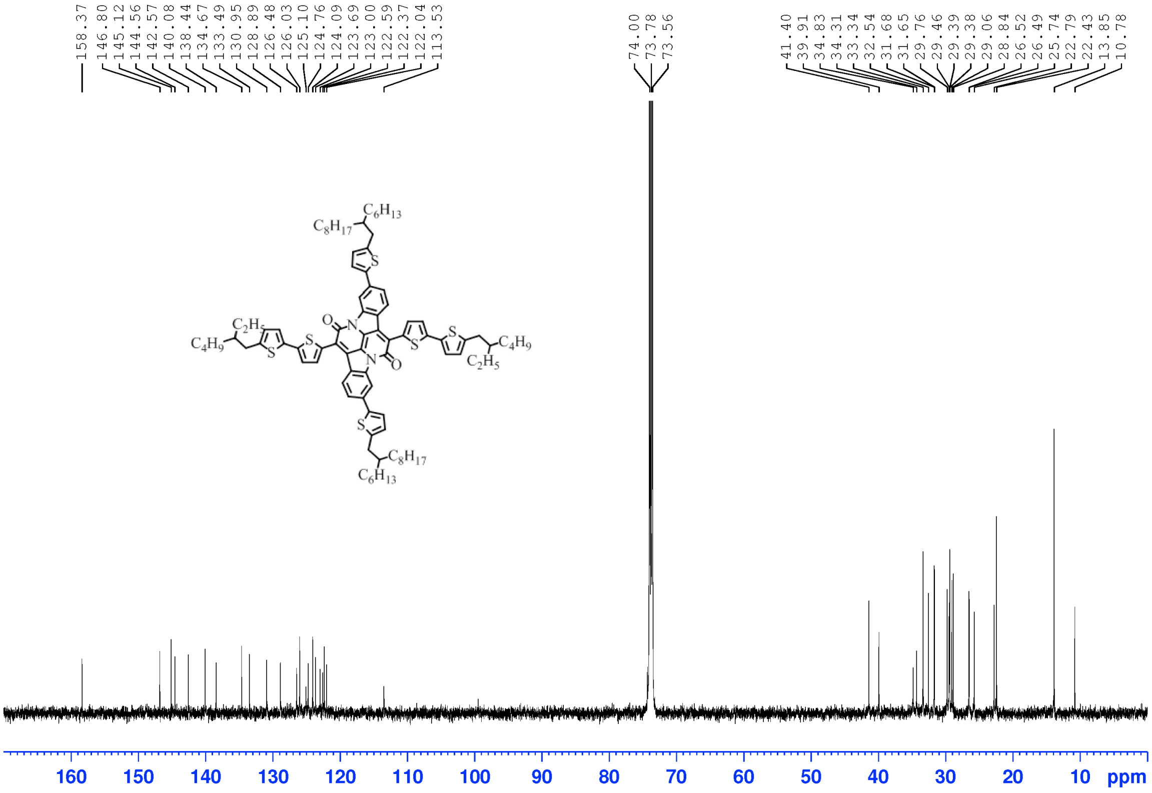

Figure S22. ^{13}C NMR spectrum of **BAI-2T$_x$-1T$_y$** ($C_2D_2Cl_4$, 353 K).

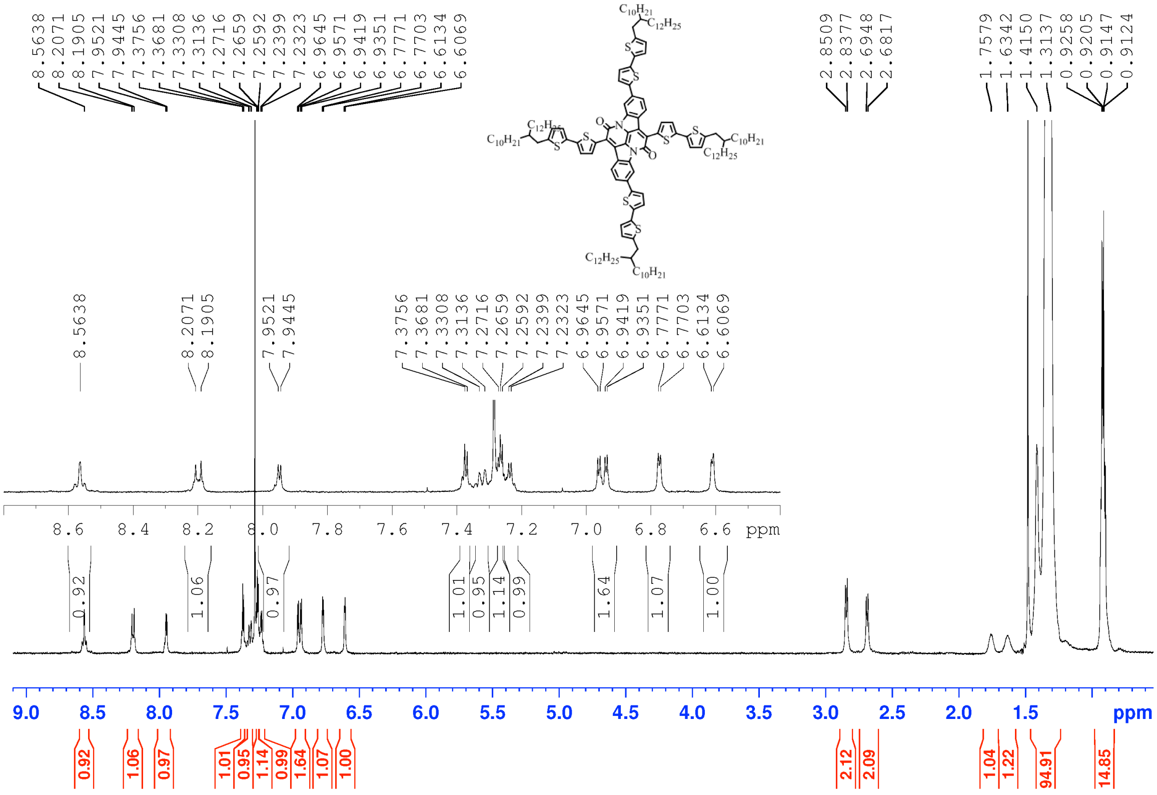

Figure S23. ^{1}H NMR spectrum of **BAI-2T$_x$-2T$_y$** ($CDCl_3$, 328 K).

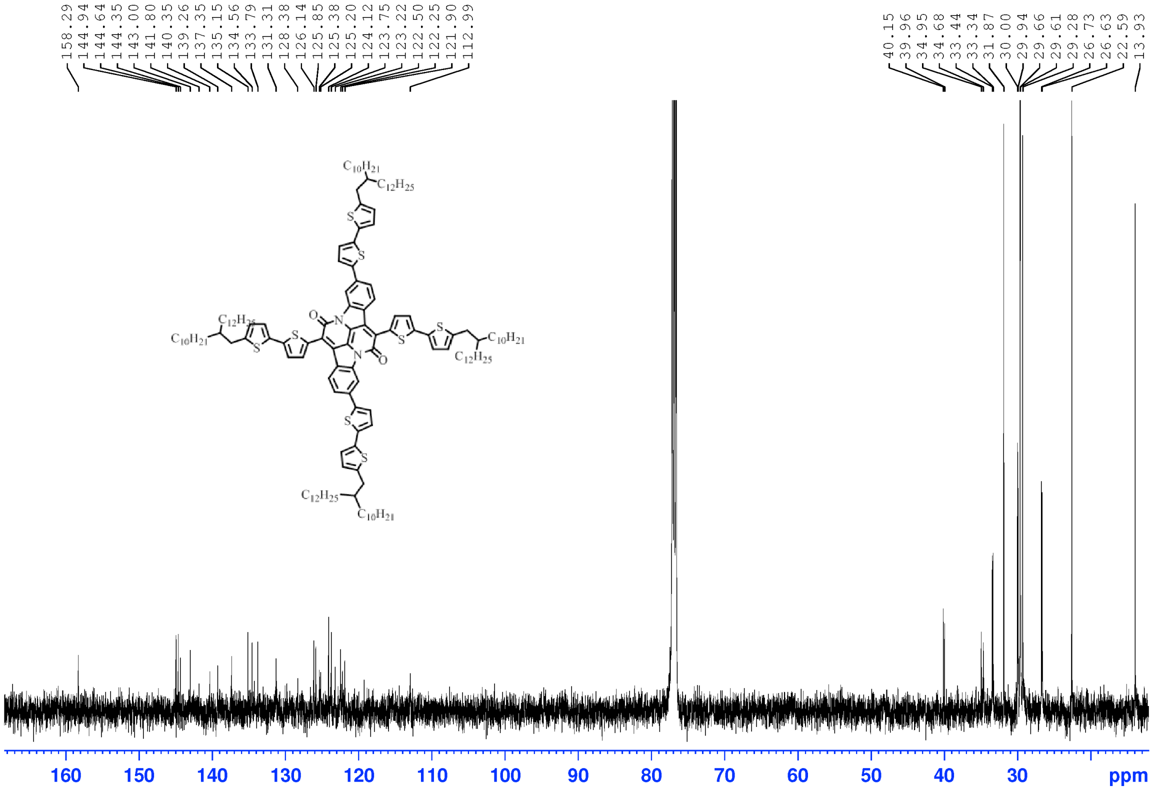

Figure S24. ^{13}C NMR spectrum of **BAI-2T$_x$-2T$_y$** (CDCl$_3$, 328 K).

References

(1) (a) Binkley, J. S.; Pople, J. A.; Hehre, W. J. *J. Am. Chem. Soc.* **1980**, *102*, 939-947. (b) Krishnan, R.; Binkley, J. S.; Seeger, R.; Pople, J. A. *J. Chem. Phys.* **1980**, *72*, 650-654.

(2) Marenich, A. V.; Cramer, C. J.; Truhlar, D. G. *J. Phys. Chem. B* **2009**, *113*, 6378-6396.

(3) Fleming, S.; Mills, A.; Tuttle, T. *Beilstein J. Org. Chem.* **2011**, *7*, 432-441.

(4) Kaur, I.; Jia, W.; Kopreski, R. P.; Selvarasah, S.; Dokmeci, M. R.; Pramanik, C.; McGruer, N. E.; Miller, G. P. *J. Am. Chem. Soc.* **2008**, *130*, 16274-16286.

(5) Zhan, C.-G.; Nichols, J. A.; Dixon, D. A. *J. Phys. Chem. A* **2003**, *107*, 4184-4195.

(6) He, B.; Pun, A. B.; Zherebetskyy, D.; Liu, Y.; Liu, F.; Klivansky, L. M.; McGough, A. M.; Zhang, B. A.; Lo, K.; Russell, T. P.; Wang, L.; Liu, Y. *J. Am. Chem. Soc.* **2014**, *136*, 15093-15101.

(7) Imming, P.; Imhof, I.; Zentgraf, M. *Synth. Commun.* **2001**, *31*, 3721-3727.

(8) Ellinger, S.; Ziener, U.; Thewalt, U.; Landfester, K.; Möller, M. *Chem. Mater.* **2007**, *19*, 1070-1075.
